# Mixed evidence for early bursts of morphological evolution in extant clades

**DOI:** 10.1111/jeb.13236

**Published:** 2018-01-18

**Authors:** M. N. Puttick

**Affiliations:** ^1^ School of Earth Sciences Bristol UK; ^2^ Department of Life Sciences Natural History Museum London UK

**Keywords:** body size, early bursts, macroevolution, mode, phylogenetic comparative methods, tempo

## Abstract

Macroevolutionary theory predicts high rates of evolution should occur early in a clade's history as species exploit ecological opportunity. Evidence from the fossil record has shown a high prevalence of early bursts in morphological evolution, but recent work has provided little evidence for early high rates in the evolution of extant clades. Here, I test the prevalence of early bursts in extant data using phylogenetic comparative methods. Existing models are extended to allow a shift from a background Brownian motion (BM) process to an early burst process within subclades of phylogenies, rather than an early burst being applied to an entire phylogenetic tree. This nested early burst model is compared to other modes of evolution that can occur within subclades, such as evolution with a constraint (Ornstein‐Uhlenbeck model) and nested BM rate shift models. These relaxed models are validated using simulations and then are applied to body size evolution of three major clades of amniotes (mammals, squamates and aves) at different levels of taxonomic organization (order, family). Applying these unconstrained models greatly increases the support for early bursts within nested subclades, and so early bursts are the most common model of evolution when only one shift is analysed. However, the relative fit of early burst models is worse than models that allow for multiple shifts of the BM or OU process. No single‐shift or homogenous model is superior to models of multiple shifts in BM or OU evolution, but the patterns shown by these multirate models are generally congruent with patterns expected from early bursts.

## Introduction

The adaptive radiation of morphological traits is a key part of macroevolutionary theory. In an adaptive radiation, a clade's early history is characterized by movement into new areas of morphospace, usually in response to ecological opportunity (Simpson, 1944; Schluter, 2000; Losos, 2010). This definition is distinct from early high rates of speciation: adaptive radiations are defined by the rapid acquisition of diverse morphological traits within closely related clades (Givnish, 2015). Within this framework, early bursts of morphological evolution are modelled on phylogenetic trees by having high early rates of change that slow exponentially through time (Simpson, 1944; Blomberg *et al*., 2003; Harmon *et al*., 2010).

Phylogenetic analyses of trait evolution have shown that early bursts are not a common feature in living groups (Cooper & Purvis, 2010; Harmon *et al*., 2010), but are not entirely absent (Harmon *et al*., 2003; Burbrink & Pyron, 2010; Slater *et al*., 2010; Derryberry *et al*., 2011). Some methodological issues may cloud the detection of early bursts in extant clades (Slater *et al*., 2010; Slater & Pennell, 2014), but their prevalence in living groups is still equivocal. However, patterns of morphological evolution are more widely recognized in the fossil record where the theory of early bursts was first formulated (Foote, 1994; Wagner, 1997; Hughes *et al*., 2013).

Here, I relax the assumption that early bursts must occur on entire phylogenies. Previously, early burst models have been applied to whole phylogenies representing traditional taxonomic groups (Harmon *et al*., 2010), although a similar relaxation of clade rates is also available in BAMM (Rabosky *et al*. 2013; Rabosky, 2014). Models of Brownian motion (BM) rate homogeneity (Felsenstein, 1973, 1985) have previously been extended by allowing nested clades to have different rates (O'Meara *et al*., 2006; Thomas *et al*., 2006), and evolutionary modes (Ingram & Mahler, 2013; Mahler *et al*., 2013; Uyeda & Harmon, 2014; Khabbazian *et al*., 2016). Here, I implement a similar approach to model nested early bursts in subclades of the phylogeny against background BM process. Additionally, I test the relative fit of these models against similar models of evolution that constrain traits to an optimum value (Hansen, 1997; Mahler *et al*., 2013; Pennell *et al*., 2015), and to models that allow for a different rate of evolution in nested clades (O'Meara *et al*., 2006; Thomas *et al*., 2006).

Using these models, I test the prevalence of nested early bursts in body size evolution in three speciose clades of extant amniotes: mammals, aves and squamates. There has been little previous evidence for early bursts within mammals overall (Cooper & Purvis, 2010), but some support in subclades of mammals, birds and squamates (Harmon *et al*., 2003; Slater *et al*., 2010; Derryberry *et al*., 2011; Slater, 2015). All models were applied to phylogenies of mammals, birds and squamates, at the order and family level. At these traditional levels of organization, there is limited evidence for early bursts. When the assumptions are relaxed so early bursts models can occur in nested subclades within these phylogenies, early burst patterns become more prevalent. However, the relative fit of these models is worse than that of models that allow for multiple shifts (> 1) in a BM or OU process, but there is evidence to indicate these models still possess signals expected from an early burst pattern.

## Materials and methods

### Models

**Figure 1:**
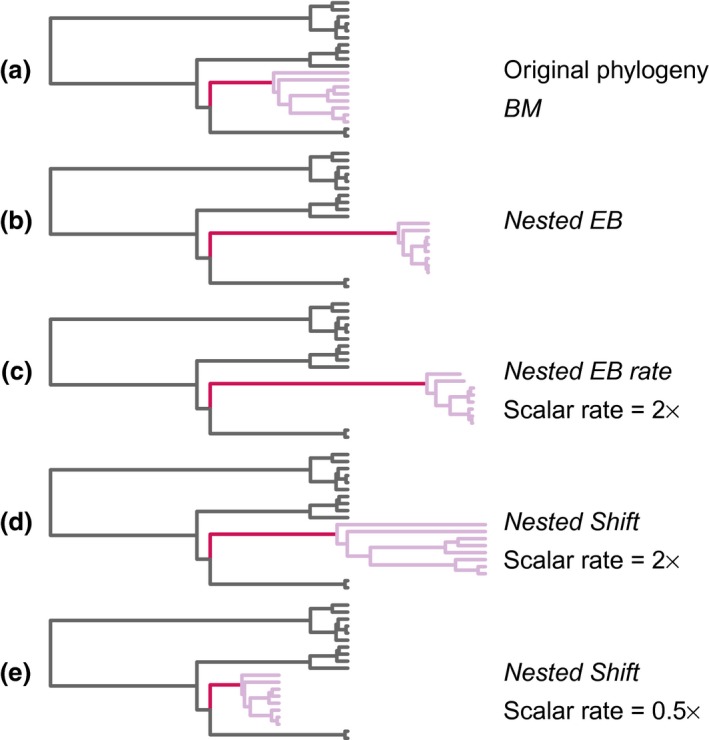
Comparison of the tree branch length transformations performed by the *BM* (a), *nested EB* (b), *nested EB rate* (c), *nested shift* rate increase (d) and *nested shift* rate decrease (e) models. For each phylogeny, it is assumed that there is change from the ancestral BM (dark branches) in a nested clade (coloured branches). No transformation occurs in the BM model (a). In the *nested EB* (b) and *nested EB rate* (c) models, there is an exponential increase on the branch leading the most recent common ancestor of the nested clade (red branch) followed by an exponential slowdown (pink branches). In the *nested EB* model, this exponential change is relative to the ancestral Brownian rate (b), and the exponential change in the *nested EB rate* is relative to the ancestral Brownian rate multiplied by a scalar. These *nested EB* models are distinct from the *nested shift* models: in the *nested shift* models, there is a linear increase (d) or decrease (e) applied to all branches with no slowdown or increase in rate, respectively.

Here, I extend previous models of early bursts (EB) so that they can occur in nested monophyletic clades within a phylogeny. This nested EB process is set against an ancestral BM model which describes the evolution of traits for species outside of the nested monophyletic clade. Specifically, I apply the two models of nested early bursts: the *nested EB* model in which the early burst process inherits the basal BM rate; and the *nested EB rate* model that is similar to the *nested EB* model except a scalar also allows for a higher rate of evolution within the nested clade compared to the ancestral rate of BM evolution. Both the *nested EB* and *nested EB rate* models allow for early increases in the rate of evolution at the base of a clade: the branch leading to the most recent common ancestor of the nested clade undergoes an increase in rate compared to the background BM rate in both the *nested EB* and *nested EB rate* models. This increase is then followed by an exponential slowdown in both the *nested EB* and *nested EB rate* models. In the *nested EB* model, the increase and slowdown is relative to the ancestral Brownian rate, but in the *nested EB rate* model, the rate increase and exponential decrease is relative to the rate scalar applied to this clade (Fig. 1).

I compare these nested models to similar models that have been previously implemented: nested models of the Ornstein‐Uhlenbeck (OU) model process (Ingram & Mahler, 2013; Uyeda & Harmon, 2014; Khabbazian *et al*., 2016); nested models in which the rate of BM can change throughout a phylogeny (O'Meara *et al*., 2006; Thomas *et al*., 2006); and models of BM, OU and EB applied to the whole phylogeny. Nested OU and nested BM models are not novel, but for consistency within the manuscript, they are designated as *nested OU* and *nested shift*. In the nested OU model, a monophyletic subclade inherits the basal BM rate, but is constrained to an optimum value by the attraction parameter (*α*) and collapses to BM when *α* = 0; and in the *nested shift* model, a monophyletic subclade is characterized by increased or decreased rates, and this is equal to BM when the nested clade has rate 1. The relative fit of models is judged by estimating the small‐sample Akaike Information Criterion (AICc) (Burnham & Anderson, 2004). All models are used to find a maximum of one shift. In an extension of this model, it would be possible to model a greater number of shifts, but this may not be appropriate with an AIC‐based approach (Ho & Ané, 2014; Khabbazian *et al*., 2016).

### Nested early bursts

The nested EB models are modifications of the widely used BM model of trait evolution (Felsenstein, 1973, 1985; Hansen, 1997; Blomberg *et al*., 2003; Harmon *et al*., 2010). Harmon *et al*. (2010) introduced the EB model in which rates exponentially slow through time as a modification of the models introduced by Blomberg *et al*. (2003). In the models presented here, nested clades can undergo an early burst in which the branch leading to the most recent common ancestor of a clade undergoes an increase in rate compared to the background and the subsequent crown clade experiences a decrease in rate.

To calculate likelihood under the BM process, it is necessary to estimate the rate parameter *σ* ^2^ and the phylogenetic mean *μ* using maximum‐likelihood estimation or by phylogenetic independent contrasts (Felsenstein, 1985; Freckleton, 2012). The likelihood of the traits given the phylogeny of ***n*** tips can then be given by eqn (1):

(1) $$\ln\left(L\right)=-\frac{1}{2}\left(n\log\left(2\pi \sigma^{2}\right)+\frac{{\left(y-\hat{\mu }X\right)}^{T}V^{-1}\left(y-\hat{\mu }X\right)}{\sigma^{2}}\right),$$

where **V** is an *n *× *n* variance–covariance matrix of branch lengths shared between *n* species (tips) on a phylogeny, **X** is the column vector of 1 and *y* is the expected mean vector of the traits. In an early burst, **V** is transformed by the parameter *r*, so edge lengths, and modelled rates, reduce exponentially through time. According to Harmon *et al*. (2010), the variance–covariance matrix is modified in eqn (2):

(2) $$V_{\mathrm{ij}}=\int_{0}^{S_{\mathrm{ij}}}\sigma_{0}^{2}e^{\mathrm{rt}}=\sigma_{0}^{2}\left(\frac{e^{rs_{\mathrm{ij}}}-1}{r}\right),$$

where *S*_*ij*_ represents the branch length to be modified, *t* is the time since the origin of the phylogeny (or the origin time of the nested clade) and *r* is the early burst parameter (restricted to be lower than zero to model rates that decrease through time). In this model, the integral is calculated over the time (*t*) since the origin of the clade to the present (0). In both the *nested EB* models, the branch leading to the nested clade is considered as part of the EB process, but the decrease in rate starts at the crown node (Fig. 1). This approach allows for an increased rate compared to the background rate on the branch leading to the crown node. The start of this branch has a negative age compared to the crown node, so this edge length is increased in an early burst process.

For the *nested EB* and *nested EB rate,* a BM model starts at the root and changes to an early burst process in a nested subclade. The variance–covariance matrix of the whole phylogeny, **V**, in these models is the sum of the background BM process **V**_**0**_ and **V**_***eb***_ which represents the modified nested clade from (2). The *n *× *n* matrix **V**_**0**_ contains nonzero covariances for taxa not found in the nested clades, and all other covariances are filled as zero (including those from within the nested subclade). The *n *× *n* matrix representing the nested clade, **V**_***eb***_, has nonzero entries for covariances of taxa within the nested clade if they share branch lengths and zero entries for all other covariances. Thus, the sum of **V**_**0**_ + **V**_***eb***_ is equal to **V** which represents the variance–covariance matrix for the whole tree. The notation and approach used here follows that of Thomas *et al*. (2006), but these matrices are equivalent to those designated as **C** in Revell & Collar (2009). In the calculation of the *nested EB* model, the nested matrix **V**_***eb***_ is transformed by the maximum‐likelihood estimate of *r*, and the combined matrix (**V**) is transformed by the maximum‐likelihood estimate of *σ* ^2^.

It is possible that a *nested EB* process can be very similar to a simple clade‐wide decrease in rate (O'Meara *et al*., 2006; Thomas *et al*., 2006), which could closely mimic an exponential decrease in rate, particularly with small values of *r*. Even so, the two models are not identical. Therefore, in the *nested EB rate* model, rates can be higher than the ancestral process as the rate of the clade is increased by a scalar, and this differs from the *nested EB* model in which the nested clade inherits the ancestral rate. The Brownian variance of the process is given by eqn (3):

(3) $$\sigma^{2}=\frac{1}{n-1}{\left(y-\hat{\mu }X\right)}^{T}{\left(V_{0}+\theta V{\;}_{eb}\right)}^{-1}\left(y-\hat{\mu }X\right),$$

where the rate scalar ***θ*** allows for a simultaneous increase in the rate of evolution in the nested early burst clade – this scalar modifies the ancestral rate variance of the BM process (Thomas *et al*., 2006). This scalar has a lower limit of 1 in which the model inherits the ancestral rate variance (i.e. *nested EB rate* collapses to the *nested EB* model when *θ* = 1). For the nested EB rate model, there are four parameters: the Brownian rate, the phylogenetic mean, the scalar and the parameter *r*.

Shifts are fit to all nodes on the phylogeny that are ancestral to clades with at least n species. The final model is chosen by identifying the node that produces the lowest AICc score. One issue is that this approach can lead a high type 1 error rate (Alfaro *et al*., 2009; Thomas & Freckleton, 2012; May & Moore, 2016). To alleviate this, I estimated the type 1 error rate with 1000 data sets simulated under BM and found the necessary AICc cut‐off to lower the final model error rates to 5%. This correction is idiosyncratic to the data so must be performed for each analysis as the use of general cut‐offs has been shown to be inadequate in similar models (May & Moore, 2016).

### Implementation

I implemented this model in custom written code in R (R Core Team, 2016) using maximum likelihood (available on GitHub, https://github.com/PuttickMacroevolution/cladeMode). Optimization of model parameters was achieved using the function *optim* available in the base *stats* package in R. Parameters were optimized by supplying upper and lower bounds of values using the method L‐BFGS‐B. The starting parameter for the BM rate was set as the variance of character trait divided by the age of the clade, and bounds of 1e‐8 and 20 were used in the parameter search. Identical parameters were used for the rate scalar parameter. A value of −0.01 was used for the EB parameter r with an upper bound of −1e‐6 and a lower bound of ln(1e‐5)/age of clade. The attraction parameter *α* was optimized in the OU models with a starting value of 0.05 with a lower bound of 1e‐8 and upper bound of 2.71. For each parameter search, a single run was used with 100 iterations.

### Simulations

To test the performance of models, I ran a series of simulations in R. The simulations were used to judge the ability each model to differentiate between different scenarios of evolution. To this end, data and trees were simulated, and then, the data sets were tested under each model considered in the study (*BM, EB, nested EB, nested EB rate, nested OU and nested shift*). I simulated birth–death (*λ* = 1, *μ* = 0.5) ultrametric trees with 50, 100, 200 and 500 tips using the R package TreeSim (Stadler, 2011). All trees were then scaled to unit length.

A range of simulation parameters were set in each analysis, with basal rate of BM (*σ* ^*2*^) of 1. In models of early burst, the upper bound of the parameter of exponential decrease (*r*) is generally set to ln (1e‐5) divided by the age of the root (1 in the unit length trees). In all EB simulations (*EB*,* nested EB, nested EB rate*), the maximum value of *r* was set to ln(1e‐5)/1, and in separate simulations, a range of smaller parameter values were based on products of this maximum value (1x, 0.95x, 0.75x, 0.5x, 0.25x and 0.05x the maximum *r*). In the *nested EB* and *nested EB rate* models, the shift node was selected at random from nodes that were ancestral to at least 25% tips of the phylogeny. For the *nested EB rate* model, the concurrent shift in the rate of BM (using the scalar ***θ***) was set to 2x, 5x and 10x the underlying BM rate, and data were simulated under the full range of *r* values for each rate shift value, respectively. Nested EB models were also assessed on ability reconstruct evolution when the model is violated. In these simulations, the shift node was selected from nodes that are ancestral to < 25% tips of the phylogeny, but the model search based on these simulated data only considered nodes that were ancestral to 25% and above.

To test for type 1 errors in the two nested EB models, data were simulated using *EB* (whole phylogeny), *OU, nested OU* and *nested shift* models. For the *OU* and *nested OU* models, the maximum value of the attraction parameter (*α*) was set to *exp(1)*, and six alternative values based on this value were used in the simulations (1x, 0.95x, 0.75x, 0.5x, 0.25x and 0.05x the maximum value of *α*). The values for the rates of evolution within nested clade with the *nested shift* model were based upon the same values in the *nested EB rate* model (2x, 5x and 10x the original rate).

### Empirical data

I used published data from three major clades of extant amniotes: mammals, birds and squamates. I applied the models to extant squamates (Title & Rabosky, 2016; Zheng & Wiens, 2016), mammals (Bininda‐Emonds *et al*., 2007) and birds (Jetz *et al*., 2012). Body size data were taken from the amniote comparative database (Myhrvold *et al*., 2015). Body size is a biologically informative trait that is known to correlate with a large number of ecologically and physiological characters (Peters, 1983), so evidence of early bursts in body size shows evidence of an adaptive radiation (Ingram *et al*., 2012).

Zero‐length branches in the Bininda‐Emonds *et al*. (2007) mammal phylogeny may affect model inference. For example, it may favour an OU process by mimicking the expected tree shape when shared history is destroyed (Cooper *et al*., 2016), or it could favour an early burst by focusing change on a branch preceding a zero‐length branch, as no change is possible on a zero‐length edge. However, this is not an issue with the squamate and avian phylogenies, and cut‐offs were performed on all trees to reduce potential errors, including those from branch lengths.

### Empirical analysis

I applied *BM, OU*,* EB*,* nested shift*,* nested OU*,* nested EB* and *nested EB rate* models individually to orders (Mammalia, Aves), and individual families that contained at least 100 species (Mammalia, Aves, Squamata). I selected phylogenies with 100 species as the simulations indicate that they are large enough to accurately capture the process of evolution, and this is a common size of data sets in comparative phylogenetics (Chira & Thomas, 2016). Overall models were fit to 43 clades that including seven mammal and 10 bird orders, and eight mammal families, 14 bird families and four squamate families, respectively. As these data represent both families and orders, each data set is not necessarily independent, as the same species can appear in two different analyses. The adequacy of different models was tested by comparing the significance of six different metrics of model adequacy using the R package Arbutus (Pennell *et al*., 2015).

### Models that allow for multiple shifts

The models considered here allowed for up to one *nested shift* only, so it is possible comparable models that allow for more shifts produce a superior model fit. I applied a multirate BM model that allows for different rates within nested clades using the *auteur* model in *geiger* (Eastman *et al*., 2011; Pennell *et al*., 2014), and a model that allowed for multiple optima in an OU process (Khabbazian *et al*., 2016). The *ℓ1ou* model was fit using the *ℓ1ou* package with up to 10 shifts in optima detected using the phylogenetic lasso method (Khabbazian *et al*., 2016). This model differs from the *nested OU* model which allows the attraction parameter *α* to be applied in nested clades, whereas the ℓ*1ou* method sets a tree‐wide rate of *α* and estimates different trait optima within nested clades. I calculated the AICc for the *ℓ1ou* model and for the *auteur* model using the maximum *a posteriori* (MAP) model from the post‐burn‐in MCMC run.

## Results

### Simulations

**Figure 2:**
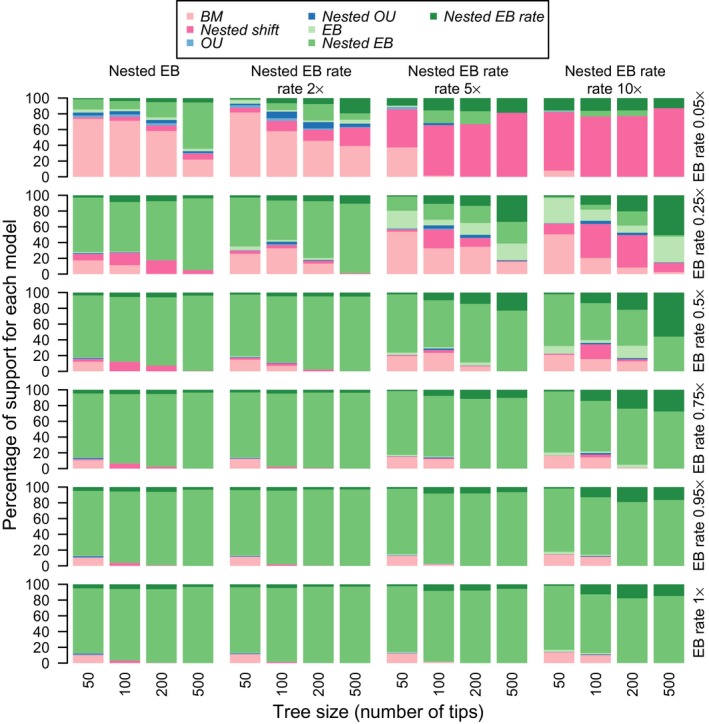
Simulation results showing the relative support for each model (as judged by AICc values) when data are simulated under the *nested EB* and *nested EB rate* models. With each model, the results are summarized when data were simulated with different values of the EB parameter *r* (0.05, 0.25, 0.5, 0.75 and 1x the maximum rate), and for *nested EB rate*, data were also simulated with a concurrent shift in BM rate 2, 5 and 10x the background rate, respectively. The *nested EB* model receives high support when it is the correct model, and tends to have higher support with data generated under the *nested EB rate* model. However, most support is for one form of the EB model (*nested EB* and *nested EB rate*).

There is generally high accuracy of models in the simulations, and accuracy increases with phylogeny size (Fig. 2). Accuracy was measured by the numbers of times the true model has the best relative AICc score. The error with the BM simulated data is improved substantially when model AICc scores are penalized to correct type 1 errors (Fig. S1). For all further simulation and empirical analyses, these corrected BM AICc values are used to judge the relative fit of models.

There is good power of the *nested EB* model on the simulation data, and support for the correct *nested EB* model increases with tree size and parameter values (Fig. 2). There is over 80% support for the correct *nested EB* model with parameter values of 0.5x, 0.75x and 1x the maximum rate of *r* on all trees (Table S1). When the *nested EB* and *nested EB rate* models are considered together, there is over 95% support at the highest parameter value (1x) on trees of 100 tips and more. There is a high level of accuracy for the estimation of the early burst parameter *r* with the *nested EB* model (Fig. S2a).

The *nested EB* model receives higher support than the *nested EB rate* model when data are simulated under both *nested EB* and *nested EB rate* models, but the highest support is for one form of the *nested EB* models. With data generated under the *nested EB rate* model, the parameter estimates for the early burst parameter *r* are lower than the true value in the *nested EB rate* model (Fig. S2b–d). The support for both *nested EB* models combined is over 95% for higher parameter values in trees with 100 tips and above (with one exception – highest shift rate (10x) on the tree with 100 tips).

The *nested EB* models have no support when model assumptions are violated. When shifts were simulated on nodes smaller than 25% of tips on the phylogeny, the *nested EB* model receives minimal support (median 5.6% support for the correct model) (Figs S2 and S3).

There is an acceptable level of error for the two *nested EB* models with data simulated under the *EB*,* OU* and *nested shift* models (Tables S2 and S3). Erroneous support for the *nested EB* models increases with data simulated under the *nested OU* model. Most of the erroneous support is for the *nested EB rate* model, as support for the *nested EB* model is under 5% for all parameter values on trees with 100 tips and above (Table S3). A similar pattern is seen with data simulated under the *nested shift* model.

### Amniote orders

**Figure 3:**
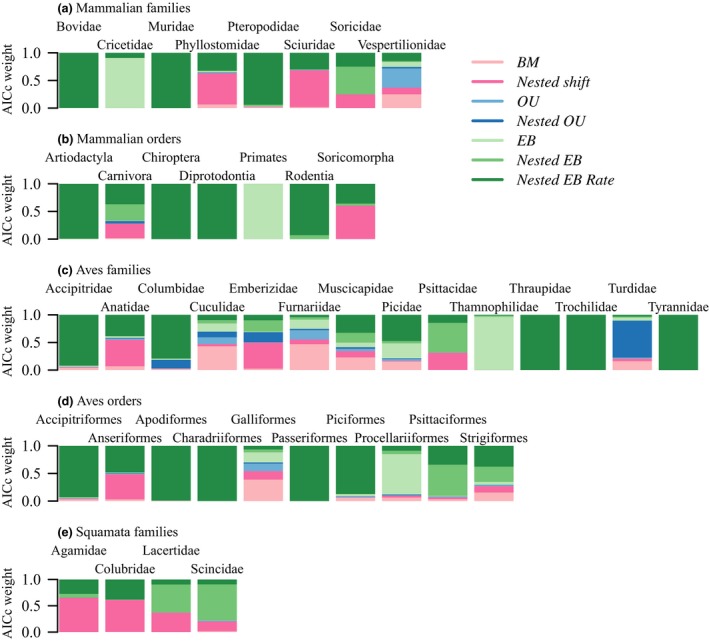
Relative support for models of body mass evolution in mammalian, avian and squamate orders and families. The bar plots represent the Akaike small‐sample weights (AICcW) of different models (*BM*,* nested shift*,*OU*,* nested OU*,*EB*,* nested EB*,* nested EB rate*) of evolution within each clade.

**Figure 4:**
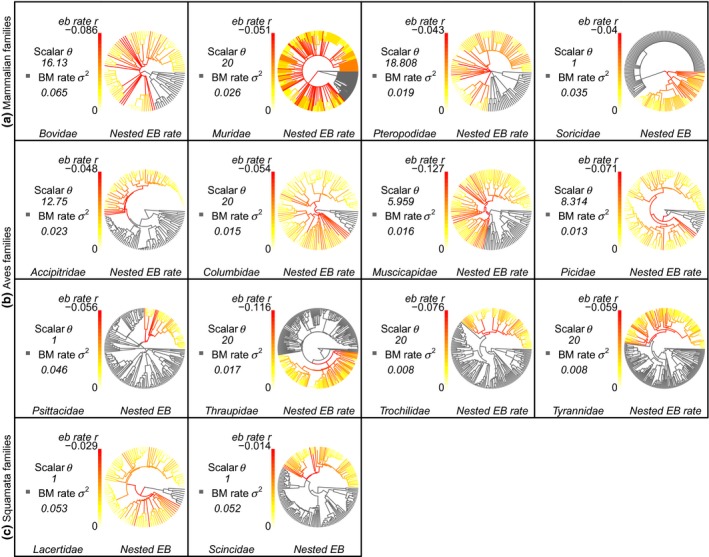
Rates of evolution in body size evolution show patterns of nested early bursts in families of Mammalia (a), Aves (b) and Squamata (c). The phylogenies show the location and pattern of nested early bursts in families in which a nested EB model (*nested EB* or *nested EB rate*) has the highest relative support. These clades have an initial high rate that eventually falls below the background rate through time. Branches are coloured to reflect the relative rates of evolution that occur in these clades in the form of the background BM rate (*σ* ^2^ – in grey), and the modification to this rate through time by the early burst parameter (*r*) and the rate scalar (*θ*).

When homogenous models are fit to the whole phylogeny, BM (33 clades) is the most widely supported model compared to EB (9 clades) and OU (1 clade). This pattern changes when homogenous models are considered alongside nested models of evolution as one form of EB is the most supported model (31/43 clades) in all analyses (Figs 3 and 4). Of these 31 clades supporting early bursts, the majority show support for *nested EB rate* (22 clades) compared to support for *nested EB* (5 clades) and *EB* (4 clades). Full parameter values are shown in the supplementary materials (Tables S4–S6).

Across all analyses, the relative performance of the different models was compared after applying AICc corrections to avoid type 1 errors; these AICc corrections were applied to each model individually. A potential source of bias may be that some models are overpenalized whereas others (e.g. EB models) are not. However, if the AICc correction is not performed across all clades, one form of the EB model is still favoured: 26/43 support one form of the EB model compared to 31/43 of clades supporting the EB model when there is no AICc correction (Table S7).

When models are applied to the order level, nested early burst shifts are only found at the base of one recognized, named family. Only Trochilidae within Apodiformes (*nested EB rate* model) shows congruence between taxonomic rank and model selection (Table S8).

### Model performance

Model adequacy improves when models are fit to smaller phylogenies representing a few hundred species. When models are fit to avian and mammalian orders, only 4/17 models are adequate according to all six metrics (Table S9). Models are prone to fail on modelling rate heterogeneity throughout the data (*Cvar*), but perform well on other metrics such as estimated overall rate (*Msig*; Table S9). Similar patterns are seen when models are applied to family data of birds and mammals (7/26 clades are fully adequate). Of all of the 11 models shown to be adequate across all metrics, six are *nested EB rate,* two *nested shift*, one *nested OU* and two *OU*.

### Multiple‐shift models

Multiple‐shift BM and OU models both respectively provide a superior fit compared to the best‐fitting nested model (Table 2). Only three of the best‐fitting nested models are superior to the multirate BM *auteur* model, and only three models are a superior fit to the ℓ*1ou* model.

The node height test can be used to test whether these multirate models show patterns expected of early bursts (Freckleton & Harvey, 2006; Slater *et al*., 2010; Slater & Pennell, 2014). The node height test is a linear model of the absolute phylogenetic independent contrasts from each internal node in a tree against the distance in time of each contrast node since the root (Freckleton & Harvey, 2006). A significant negative slope (higher disparity near the root of the clade) can be interpreted as an EB pattern. The expected contrasts were calculated for the median branch rates from the multirate BM *auteur* model, and the multirate OU model branch parameters from the *l10U* analysis. As with Slater & Pennell (2014), the log of absolute contrasts was used to model exponential decreases through time. Phylogenies were pruned to include only those taxa in clades marked by an early burst process when using the *nested EB* and *nested EB rate* models. Nineteen of the 27 clades that support a *nested EB* model show a negative slope when using contrasts from the multi‐BM model in the node height test (Table S10), and 20 clades show a negative slope with contrasts from the multi‐OU model. These negative slopes are significant for 11 of the multirate BM models and 9 OU models, respectively. Outliers can affect estimation of early burst patterns through time (Slater *et al*., 2010; Slater & Pennell, 2014), so the analyses were repeated by removing contrasts with values greater than or less than 3 standard deviations from the mean and using a robust linear regression model (Slater & Pennell, 2014). After outliers were removed, eight of the multirate BM models and nine OU models showed a significant decrease in absolute contrast values through time (Table S10). Using the robust linear regression, 18 multirate BM and OU models show a negative decrease in the value of contrasts through time.

Some of the patterns of rates through time shown by the multirate BM model are congruent with those from the *nested EB* and *nested EB rate* models. Analyses from *auteur* show a shift in rate on the same branch as indicated by either *nested EB* or *nested EB rate* models in 10 of the clades that support these two nested EB processes (Table S10). Furthermore, for clades that support either *nested EB* or *nested EB* rate, we would generally expect the estimated median branch rates from *auteur* in the EB clade to be lower than the non‐EB rates in the rest of the phylogeny, and for the crown branch leading to the EB clade to have a rate higher than the rates within the EB clade. These two measures are significant (*Wilcoxon test*) for 11 of the 28 clades that show the highest relative support either *nested EB* or *nested EB rate* (Table S11).

## Discussion

Previous evidence for early bursts of morphological evolution in extant species has been equivocal, but here I show that early bursts are more frequently detected in living mammal, bird and squamate clades when applied to nested clades. When applied to phylogenies representing traditional taxonomic groups – order and family – there is little support for early burst patterns. When this taxonomic constraint is lifted, there is a strong, general signal for the pattern of early bursts. The increased detection of early bursts may partly reflect the lifted constraint of rates being applied to named clades, and by allowing for the modelling of processes that occur on branches leading to extant clades. However, the increased support for nested early bursts is only seen when comparing the relative fit of homogenous or single‐shift models. This increased support for early bursts is not seen when compared to models of rate heterogeneity that allow for multiple shifts in a BM or OU process (Table 2). No model with homogenous evolution or a single shift is superior to models with general rate heterogeneity (Table 2), but the patterns of change shown by multirate models are generally congruent with patterns expected from an early burst (Table S10).

### Early bursts

Although multiple shifts perform better than single‐shift models, early bursts are the most common process when only a maximum of a single shift is allowed (Table S2). Evidence for a high prevalence of early bursts in extant phylogenies brings congruence between the previously contrasting conclusions on the relative occurrence of early bursts from analyses from the fossil record and living species. Much of the early theory of early bursts (Simpson, 1944), and more recent evidence (Foote, 1994; Wagner, 1997; Hughes *et al*., 2013), comes from analyses in the fossil record. One potential reason for this difference is that analyses in the fossil record generally focus on processes of disparity whereas extant studies use rates analyses. Furthermore, in fossil data there is evidence of shifts being confined to subclades and being separated in time (Wagner, 1997), and a similar pattern is found here.

It could be argued here that the patterns here do not conform to classic adaptive radiations as they apply to large phylogenies, and are not confined to named Linnaean taxonomic clades. There is a large amount of dispute about what constitutes an adaptive radiation (Schluter *et al*., 1997; Losos, 2010; Pincheira‐Donoso *et al*., 2015), but the most accepted definition indicates that it is a pattern in which clades undergo high morphological evolution early in their history (Schluter, 2000; Givnish, 2015). Here, I model this process but allow it to not be constrained to traditional taxonomic ranks. However, this still fulfils the definition of an adaptive radiation model as a monophyletic group undergoes a high early rate of evolution early in its history followed by a decrease in the rate (Schluter, 2000). The only difference to previous models is the choice of group: in the past, early burst models were usually applied to arbitrary taxonomic groups (Harmon *et al*., 2010), but by relaxing this constraint, I find early bursts in many named or unnamed monophyletic groups (Figs 3 and 4). Another issue may be that the definition of an early burst related as a rate that exponentially decreases through time may not capture an adaptive radiation process. The modelling of early bursts in the way used here and described previously (Harmon *et al*., 2010) may only apply to a single definition of adaptive radiations, but this definition may only signify an early burst rather than an adaptive radiation.

The two nested EB models perform generally well under simulations, but the *nested EB rate* model generally has a quite high type 2 error rate for data simulated under the *nested shift* and *nested OU* models (Table S3). A potential reason for the high type 2 error rate with the *nested EB rate* model is its ability to vary both the rate and the early burst parameter *r* to explain trait evolution (Fig. S2), and the poor general power of models to recapture OU processes (Cooper *et al*., 2016). Yet it is unlikely all support for the *nested EB rate* model in the empirical data set is due to errors. For example, in all data sets the two processes for which *nested EB rate* showed high type 2 errors – *nested OU* and *nested shifts –* are the second‐best model in 9/22 clades when *nested EB rate* is the best relative‐fitting model. Even if *nested EB rate* was incorrectly supported over *nested OU* and *nested shift* in all these cases, there are still 20 clades that support some form of early burst process. Furthermore, in simulations the parameter space in which the *nested EB rate* model shows the most power is when data are simulated with high scalar rates (*θ* around 10x the background rate) and relatively low values (around 0.25–0.5x the maximum rate) of the rate parameter *r* (Fig. 1). In the empirical data (Tables S4–S6), when there is support for the *nested EB rate* model, parameter values for *θ* and *r* are in this region of high scalar rates and low values of *r*: the estimated value of *θ* is at least over 5x the background rate in the majority of clades that support the *nested EB rate* model, and these clades also show lower values of *r* (the estimate of *r* is less than 0.5x the maximum rate). These results suggest that support for the *nested EB rate* model is not erroneous when the relative fit of homogenous and single‐shift models is compared.

Recent analyses have indicated how the use of OU models on clades of a small size can lead to elevated type 1 error rates and difficulties in interpretations (Cooper *et al*., 2016). Thus, the use of OU models in the analysis can lead to similar problems. However, there is no attempt here to interpret the biological meaning of the OU models, and there is little support for OU models generally (Table 1). It is always important to carefully to adjudge comparative methods (Cooper *et al*., 2016), but the use of OU models here does not appear to present a problem. Also, problems with the OU model are most pronounced on phylogenies with fewer than 200 tips, and 16 of the clades analysed here have more than 200 tips (Table S12).

**Table 1:** The supported models when body mass evolution is analysed at the whole‐phylogeny level (*BM*,* OU* and *EB*) compared to models when the initial mode of BM evolution can change within nested clades (*nested EB*,* nested EB rate*,* nested OU* and *nested shift*). All models were also applied individually to families, orders and suborders with at least 100 species

		Whole‐phylogeny model	AICcW	All models	AICcW
Mammalia Orders	Artiodactyla	BM	0.545	Nested EB rate	0.993
	Carnivora	BM	0.602	Nested EB rate	0.371
	Chiroptera	BM	0.603	Nested EB rate	1.000
	Diprotodontia	EB	0.931	Nested EB rate	0.999
	Primates	EB	1.00	EB	0.999
	Rodentia	EB	1.00	Nested EB rate	0.929
	Soricomorpha	BM	0.626	Nested shift	0.605
Mammalia Families	Bovidae	EB	0.650	Nested EB rate	0.997
	Cricetidae	EB	1	EB	0.904
	Muridae	EB	0.650	Nested EB rate	1.00
	Phyllostomidae	BM	0.667	Nested shift	0.564
	Pteropodidae	BM	0.604	Nested EB rate	0.939
	Sciuridae	BM	0.634	Nested shift	0.666
	Soricidae	BM	0.457	Nested EB	0.503
	Vespertilionidae	OU	0.507	OU	0.347
Aves Orders	Accipitriformes	BM	0.656	Nested EB rate	0.919
	Anseriformes	BM	0.587	Nested EB rate	0.481
	Apodiformes	BM	0.582	Nested EB rate	0.989
	Charadriiformes	BM	0.5831	Nested EB rate	0.999
	Galliformes	BM	0.549	BM	0.389
	Passeriformes	BM	0.606	Nested EB rate	1.00
	Piciformes	BM	0.528	Nested EB rate	0.871
	Procellariiformes	EB	0.912	EB	0.729
	Psittaciformes	BM	0.588	Nested EB	0.564
	Strigiformes	BM	0.689	Nested EB rate	0.378
Aves Families	Accipitridae	BM	0.656	Nested EB rate	0.919
	Anatidae	BM	0.587	Nested shift	0.479
	Columbidae	BM	0.524	Nested EB rate	0.791
	Cuculidae	BM	0.613	BM	0.430
	Emberizidae	BM	0.628	Nested shift	0.470
	Furnariidae	BM	0.584	BM	0.468
	Muscicapidae	BM	0.652	Nested EB rate	0.326
	Picidae	EB	0.591	Nested EB rate	0.475
	Psittacidae	BM	0.590	Nested EB	0.537
	Thamnophilidae	EB	0.999	EB	0.964
	Thraupidae	BM	0.582	Nested EB rate	0.999
	Trochilidae	BM	0.583	Nested EB rate	0.998
	Turdidae	BM	0.680	Nested OU	0.668
	Tyrannidae	BM	0.582	Nested EB rate	0.999
Squamate Families	Agamidae	BM	0.664	Nested shift	0.652
	Colubridae	BM	0.510	Nested shift	0.607
	Lacertidae	BM	0.673	Nested EB	0.533
	Scincidae	BM	0.485	Nested EB	0.685

### Rate heterogeneity

The results here support the conclusions that heterogeneity of modes is a generality of clades, and this rate heterogeneity provides superior fit to models of early bursts. The superior fit of multirate BM and OU models compared to the best‐fitting nested models, including the early burst models, indicates that rate heterogeneity and different optima are characteristic of the analysed empirical data sets (Table 2). The models here were implemented to detect a named model of evolution, such as the nested early burst, and compare the relative model fit in a simple hypothesis‐driven framework. However, as these models only allow for one shift, they are suboptimal compared to models that allow for rate heterogeneity (Table 2). These results suggest that there is a general pattern of rate heterogeneity in clades containing a few hundred species (Pennell *et al*., 2015; Chira & Thomas, 2016).

**Table 2:** A summary of the relative fit (as judged by AICc) of alternative models – a multirate Brownian motion model fit using *Auteur* and a multi‐optima OU model fit using *ℓ1ou* – compared to the best‐fitting nested models from the analyses

	Nested models	Nested model AICc	Auteur AICc	Auteur *n* shifts	*ℓ1ou* AICc	*ℓ1ou n* shifts
Artiodactyla	Nested EB rate	413.6701797	401.9048276	3	412.4176632	6
Bovidae*	Nested EB rate	247.4473073	253.4777778	4	243.5713585	5
Carnivora‡	Nested EB rate	570.8728199	549.8762115	2	541.8213844	8
Chiroptera‡	Nested EB rate	1197.783157	1155.745902	7	1160.282015	8
Cricetidae	EB	458.687641	391.4687671	6	423.6174566	7
Diprotodontia	Nested EB rate	222.8277938	205.4452174	6	197.6960485	7
Muridae	Nested EB rate	754.5487677	682.7626598	8	750.9958031	8
Phyllostomidae	Nested shift	200.6869689	181.9618803	2	182.9925077	6
Primates*	EB	174.4758496	174.9570297	3	152.0179716	6
Pteropodidae	Nested EB rate	219.9480666	217.92	2	204.2372506	2
Rodentia†, ‡	Nested EB rate	2523.241482	2373.733735	15	2581.432859	8
Sciuridae	Nested shift	483.9304195	430.0668122	4	452.7922876	5
Soricidae‡	Nested EB	330.8898721	298.1892737	4	295.4438663	9
Soricomorpha	Nested shift	403.5386465	361.5558852	5	369.510108	9
Vespertilionidae	OU	289.9693185	278.6502703	3	265.5770231	9
Accipitridae	Nested EB rate	251.3695573	245.3411765	3	233.1219722	6
Accipitriformes	Nested EB rate	279.6741831	275.6233333	4	256.6471319	5
Anatidae	Nested shift	151.4251021	132.0453435	2	129.0050349	6
Anseriformes‡	Nested EB rate	159.1046522	139.1985075	2	142.9522219	5
Apodiformes	Nested EB rate	130.5006444	95.20842105	4	115.7242365	5
Charadriiformes†, ‡	Nested EB rate	303.6593577	267.4419139	4	337.6946089	5
Columbidae	Nested EB rate	152.2631899	142.0561345	2	112.5599573	8
Cuculidae	BM	151.7609481	140.8640404	2	136.9780506	5
Emberizidae	Nested shift	25.22289734	17.6240404	2	15.85158581	5
Furnariidae	BM	128.6948998	111.2896703	3	106.6774118	7
Galliformes	BM	201.8302838	183.0031579	2	180.3622616	8
Muscicapidae	Nested EB rate	68.73925357	52.79972028	2	49.56107709	6
Passeriformes†	Nested EB rate	2280.822468	2018.57361	15	2461.958341	8
Picidae	Nested EB rate	111.4378457	106.5133333	2	101.5453547	6
Piciformes	Nested EB rate	209.298013	206.5257143	3	187.9596002	6
Procellariiformes	EB	132.4406716	122.0715789	3	88.87576231	7
Psittacidae†	Nested EB	262.1268752	237.1444444	4	250.9552026	6
Psittaciformes	Nested EB	308.0178077	280.7884615	3	292.897804	6
Strigiformes	Nested EB rate	154.9907349	111.833617	4	130.936671	7
Thamnophilidae*	EB	37.01375737	37.37666667	3	24.73317545	4
Thraupidae	Nested EB rate	150.1047229	97.91939163	4	135.7770833	7
Trochilidae	Nested EB rate	66.53074821	34.89411765	3	57.32597617	6
Turdidae	Nested OU	1.341658919	−17.17904762	2	−33.83028026	7
Tyrannidae‡	Nested EB rate	134.7737292	80.368	5	108.9193106	6
Agamidae	Nested shift	285.9825451	273.9373585	2	270.397899	8
Colubridae	Nested shift	381.090453	369.8709735	3	369.9102853	6
Lacertidae‡	Nested EB	225.5602935	201.7807477	3	168.2160774	8
Scincidae‡	Nested EB	544.2988656	528.7714917	3	515.0596078	7

Only a handful of the best‐fitting models from the nested model are superior to the best‐fitting *auteur* model (signified by *) and the best‐fitting *ℓ1ou* model (signified by †). No nested model is superior to both of these alternative models.

‡These clades show a rate shift at the same branch as the branch indicated by the supported *nested EB* or *nested EB rate* model.

The general patterns shown in the multirate OU and BM models are similar to those expected under an early burst model. Analyses of the contrasts for the multi‐BM and multi‐OU models in a node height test indicate that there is a pattern of decreasing contrast values through time in subclades that support *nested EB* or *nested EB rate* models (Table S10). This is the expected pattern of early bursts (Freckleton & Harvey, 2006; Slater *et al*., 2010) and is relatively unchanged by the presence of outliers (Slater & Pennell, 2014). Even though the two multirate models are not named early burst processes, they still retain signals expected of early bursts.

The models used in this study are designed to test the prevalence of early bursts in extant data. These models are not presented as alternatives to existing software, especially for analysis on large phylogenies that allow for multiple shifts. For example, the BAMM software implements a pattern in which rates of Brownian evolution can also slow through time (Rabosky, 2014), and a similar model applied to mammals has shown patterns of high rates leading to clades followed by a slowdown (Eastman *et al*., 2011; Venditti *et al*., 2011).

In the future, the models presented here could be extended to allow for more than one nested shift in the *EB* model and other processes; this would allow for a fairer comparison to the multiple‐shift BM and OU models. In the nested models presented here, there is only a single shift in the mode of evolution: an ancestral BM model is replaced by a new model in a nested clade. These models can be extended to allow for multiple shifts, shifts within other nested shifts, and with different modes other than BM as the ancestral process. However, when there are multiple shifts in a phylogeny, the implementation of an AICc selection procedure is inappropriate and can lead to issues of nonidentifiability (Ho & Ané, 2014). For multiple shifts, alternative methods of model selection will be preferable, such as the phylogenetic lasso method (e.g. Khabbazian *et al*., 2016) and reversible‐jump MCMC methods (Rabosky, 2014).

## Conclusions

The results here present a mixed picture. It is possible to detect a higher number of early burst processes when they are not confined to whole phylogenies, but this higher prevalence is not as powerful explaining trait evolution when compared to models that allow for multiple shifts in the underlying process. Generally, trait evolution is a process that is best explained by multiple modes and rate heterogeneity (Venditti *et al*., 2011; Chira & Thomas, 2016), and in the future models, similar models could incorporate multiple shifts in the early burst process.

## Data accessibility

All R scripts used in the manuscript have been uploaded as supplementary information along with the trees and body mass data for empirical analyses. The R code can be obtained from GitHub (https://github.com/PuttickMacroevolution/cladeMode). All scripts and data are available on figshare (https://doi.org/10.6084/m9.figshare.5469583.v1).

## Supporting information


**Figure S1** Support for all models with data simulated under BM.
**Figure S2** The estimates of the early burst parameter *r* for all trees compared to the true rate (dotted black line) for the *nested EB* and *nested EB rate* models when data are simulated under each of these model respectively.
**Figure S3** Simulation results showing the relative support for each model (as judged by AICc values) when data are simulated under the *nested EB* model, but shifts are only allowed in nodes ancestral to 5% of tips on the phylogeny and below.
**Figure S4**. Simulation results showing the relative support for each model (as judged by AICc values) when data are simulated under the *nested EB rate* model, but shifts are only allowed in nodes ancestral to 5% of tips on the phylogeny and below.
**Table S1** The support for models with data simulated under the *nested EB* model.
**Table S2** The support for models with data simulated under the *nested EB rate* model.
**Table S3** The support for models with data simulated under the *EB, OU, nested OU,* and *rate shift* models.
**Table S4.** A summary of model parameters for models fit to body size evolution in mammalian clades.
**Table S5** A summary of model parameters for models fit to body size evolution in bird clades.
**Table S6** A summary of model parameters for models fit to body size evolution in squamate clades.
**Table S7** The supported models with no AICc correction when body mass evolution is analysed at the whole‐phylogeny level (BM, OU, and EB) compared to models when the initial mode of BM evolution can change within nested clades (nested EB, nested EB rate, nested OU, and nested Shift**).** All models were also applied individually to families, orders, and sub‐orders with at least 100 species.
**Table S8** A summary of the families included within the nested shift models. Families marked as *partial* indicate only a subset of the family was included in the nested shift model.
**Table S9** Model adequacy of the best relative model tested using six metrics described by Pennell *et al*. (2015).
**Table S10** Node height tests on the estimated log‐scaled standardised independent phylogenetic contrasts expected under multi‐rate Brownian motion and OU models respectively.
**Table S11** A summary of rates from the *auteur* analyses for trees that show the highest support for the *nested EB and nested EB rate* models.
**Table S12** The number of species included in each phylogeny in the analysis.
