# Supplementary material for: Mixed evidence for early bursts of morphological evolution in extant clades
Source: J Evol Biol. 2018 Jan 18;31(4):502–15. doi: 10.1111/jeb.13236 (PMC6849586; doi:10.1111/jeb.13236)
Supplement: Supplementary file 1 — Figure S1 Support for all models with data simulated under BM. Figure S2 The estimates of the early burst parameter r for all trees compared to the true rate (dotted black line) for the nested EB and nested EB rate models when data are simulated under each of these model respectively. Figure S3 Simulation results showing the relative support for each model (as judged by AICc values) when data are simulated under the nested EB model, but shifts are only allowed in nodes ancestral to 5% of tips on the phylogeny and below. Figure S4. Simulation results showing the relative support for each model (as judged by AICc values) when data are simulated under the nested EB rate model, but shifts are only allowed in nodes ancestral to 5% of tips on the phylogeny and below. Table S1 The support for models with data simulated under the nested EB model. Table S2 The support for models with data simulated under the nested EB rate model. Table S3 The support for models with data simulated under the EB, OU, nested OU, and rate shift models. Table S4. A summary of model parameters for models fit to body size evolution in mammalian clades. Table S5 A summary of model parameters for models fit to body size evolution in bird clades. Table S6 A summary of model parameters for models fit to body size evolution in squamate clades. Table S7 The supported models with no AICc correction when body mass evolution is analysed at the whole‐phylogeny level (BM, OU, and EB) compared to models when the initial mode of BM evolution can change within nested clades (nested EB, nested EB rate, nested OU, and nested Shift). All models were also applied individually to families, orders, and sub‐orders with at least 100 species. Table S8 A summary of the families included within the nested shift models. Families marked as partial indicate only a subset of the family was included in the nested shift model. Table S9 Model adequacy of the best relative model tested using six metrics described by Pennell et al. [file JEB-31-502-s001.docx]

**Supplementary Materials**


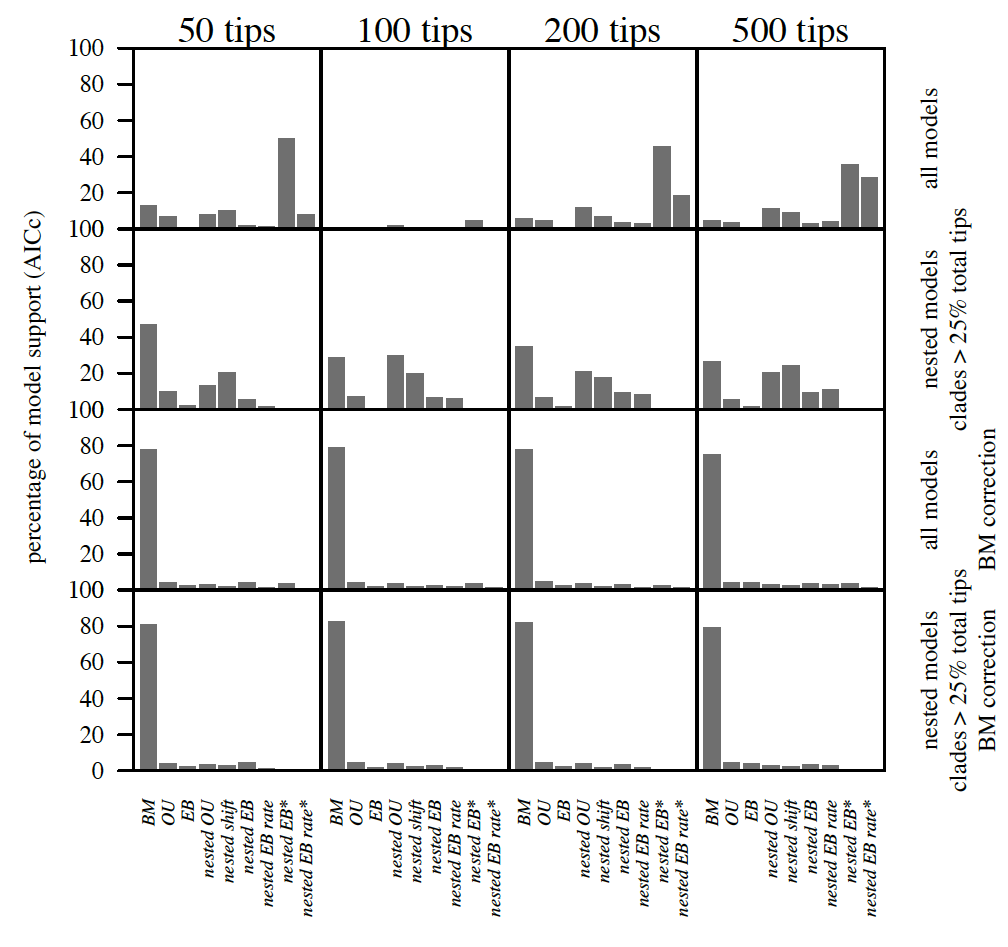


**Supplementary Figure S1.** Support for all models with data simulated under BM. There is a high type-one error rate for most models (top row), but particularly the *nested EB* and *nested EB rate* models when fit to nodes to ancestral to clades with at least 5% tips of the phylogeny (designated as *nested EB** and *nested EB rate**). There is lower type-one error when these models are excluded and the *nested EB* and *nested EB rate* models are set to clades that represent 25% of the phylogeny (second row). The lowest type-one errors are shown when the *nested EB* and *nested EB rate* models are set to clades representing 25% of the phylogeny, and a BM AICc correction is applied.

|  | ***EB rate*** | ***BM*** | ***OU*** | ***EB*** | ***nested OU*** | ***nested shift*** | ***nested EB*** | ***nested EB rate*** | ***Both nested EB models*** |
| --- | --- | --- | --- | --- | --- | --- | --- | --- | --- |
| **Tree 50** | *0.05x* | 736 | 25 | 39 | 37 | 18 | 131 | 14 | 145 |
|  | *0.25x* | 174 | 4 | 12 | 9 | 80 | 693 | 28 | 721 |
|  | *0.5x* | 122 | 5 | 6 | 11 | 33 | 787 | 36 | 823 |
|  | *0.75x* | 106 | 7 | 5 | 12 | 11 | 812 | 47 | 859 |
|  | *1x* | 99 | 8 | 5 | 11 | 4 | 822 | 51 | 873 |
|  |  |  |  |  |  |  |  |  |  |
| **Tree 100** | *0.05x* | 711 | 30 | 26 | 40 | 52 | 104 | 37 | 141 |
|  | *0.25x* | 113 | 3 | 8 | 6 | 156 | 629 | 85 | 714 |
|  | *0.5x* | 2 | 0 | 1 | 0 | 119 | 822 | 56 | 878 |
|  | *0.75x* | 0 | 0 | 1 | 0 | 65 | 880 | 54 | 934 |
|  | *1x* | 0 | 0 | 0 | 0 | 31 | 910 | 59 | 969 |
|  |  |  |  |  |  |  |  |  |  |
| **Tree 200** | *0.05x* | 582 | 29 | 33 | 41 | 70 | 194 | 51 | 245 |
|  | *0.25x* | 2 | 1 | 1 | 0 | 173 | 748 | 75 | 823 |
|  | *0.5x* | 0 | 0 | 0 | 0 | 72 | 867 | 61 | 928 |
|  | *0.75x* | 0 | 0 | 0 | 0 | 23 | 924 | 53 | 977 |
|  | *1x* | 0 | 0 | 0 | 0 | 3 | 935 | 62 | 997 |
|  |  |  |  |  |  |  |  |  |  |
| **Tree 500** | *0.05x* | 219 | 8 | 32 | 23 | 75 | 588 | 55 | 643 |
|  | *0.25x* | 0 | 0 | 0 | 0 | 50 | 912 | 38 | 950 |
|  | *0.5x* | 0 | 0 | 0 | 0 | 4 | 957 | 39 | 996 |
|  | *0.75x* | 0 | 0 | 0 | 0 | 0 | 964 | 36 | 1000 |
|  | *1x* | 0 | 0 | 0 | 0 | 0 | 967 | 33 | 1000 |

**Supplementary Table S1.** The support for models with data simulated under the *nested EB* model. Results are shown for different tree sizes, and for several different rates of the EB rate parameter (*r*). Different values of *r* were based on transformations of the maximum value used in simulations (1x, 0.95x, 0.75x, 0.5x, 0.25x, and 0.05x the maximum rate -9.21). Support for the correct nested EB model increases with tree size, and support for one form of the nested EB model increases to over 95% on trees with 100 tips at the higher parameter values.

|  | ***EB rate*** | *BM* | *OU* | *EB* | *nested OU* | *nested shift* | *nested EB* | *nested EB rate* | *Both nested EB models* |
| --- | --- | --- | --- | --- | --- | --- | --- | --- | --- |
| ***Tree 50*** | *0.05x, rate 2* | 817 | 32 | 44 | 19 | 61 | 12 | 15 | 27 |
|  | *0.25x, rate 2* | 258 | 5 | 46 | 2 | 39 | 622 | 28 | 650 |
|  | *0.5x, rate 2* | 147 | 4 | 9 | 6 | 25 | 783 | 26 | 809 |
|  | *0.75x, rate 2* | 121 | 3 | 8 | 7 | 5 | 823 | 33 | 856 |
|  | *1x, rate 2* | 111 | 3 | 7 | 8 | 2 | 831 | 38 | 869 |
| ***Tree 50*** | *0.05x, rate 5* | 373 | 24 | 18 | 4 | 484 | 1 | 96 | 97 |
|  | *0.25x, rate 5* | 538 | 5 | 227 | 6 | 28 | 181 | 15 | 196 |
|  | *0.5x, rate 5* | 195 | 3 | 29 | 0 | 11 | 739 | 23 | 762 |
|  | *0.75x, rate 5* | 148 | 4 | 17 | 1 | 5 | 807 | 18 | 825 |
|  | *1x, rate 5* | 122 | 4 | 11 | 3 | 1 | 835 | 24 | 859 |
| ***Tree 50*** | *0.05x, rate 10* | 79 | 10 | 9 | 1 | 742 | 0 | 159 | 159 |
|  | *0.25x, rate 10* | 505 | 6 | 317 | 1 | 136 | 16 | 19 | 35 |
|  | *0.5x, rate 10* | 208 | 2 | 98 | 0 | 14 | 656 | 22 | 678 |
|  | *0.75x, rate 10* | 163 | 1 | 38 | 0 | 3 | 774 | 21 | 795 |
|  | *1x, rate 10* | 136 | 3 | 27 | 0 | 3 | 813 | 18 | 831 |
| ***Tree 100*** | *0.05x, rate 2* | 580 | 34 | 21 | 83 | 131 | 85 | 66 | 151 |
|  | *0.25x, rate 2* | 328 | 10 | 23 | 33 | 40 | 500 | 66 | 566 |
|  | *0.5x, rate 2* | 69 | 0 | 4 | 7 | 33 | 839 | 48 | 887 |
|  | *0.75x, rate 2* | 2 | 0 | 1 | 0 | 21 | 928 | 48 | 976 |
|  | *1x, rate 2* | 0 | 0 | 1 | 0 | 14 | 938 | 47 | 985 |
| ***Tree 100*** | *0.05x, rate 5* | 15 | 1 | 3 | 28 | 640 | 156 | 157 | 313 |
|  | *0.25x, rate 5* | 327 | 12 | 73 | 41 | 238 | 201 | 108 | 309 |
|  | *0.5x, rate 5* | 234 | 2 | 15 | 21 | 34 | 597 | 97 | 694 |
|  | *0.75x, rate 5* | 120 | 0 | 11 | 11 | 11 | 770 | 77 | 847 |
|  | *1x, rate 5* | 14 | 0 | 2 | 0 | 2 | 899 | 83 | 982 |
| ***Tree 100*** | *0.05x, rate 10* | 0 | 0 | 0 | 0 | 766 | 72 | 162 | 234 |
|  | *0.25x, rate 10* | 205 | 2 | 141 | 39 | 430 | 63 | 120 | 183 |
|  | *0.5x, rate 10* | 156 | 1 | 36 | 18 | 186 | 469 | 134 | 603 |
|  | *0.75x, rate 10* | 142 | 0 | 19 | 20 | 36 | 642 | 141 | 783 |
|  | *1x, rate 10* | 102 | 0 | 15 | 8 | 2 | 746 | 127 | 873 |
| ***Tree 200*** | *0.05x, rate 2* | 457 | 17 | 20 | 80 | 141 | 209 | 76 | 285 |
|  | *0.25x, rate 2* | 133 | 2 | 26 | 9 | 35 | 720 | 75 | 795 |
|  | *0.5x, rate 2* | 0 | 0 | 0 | 0 | 22 | 929 | 49 | 978 |
|  | *0.75x, rate 2* | 0 | 0 | 0 | 0 | 6 | 959 | 35 | 994 |
|  | *1x, rate 2* | 0 | 0 | 0 | 0 | 1 | 968 | 31 | 999 |
| ***Tree 200*** | *0.05x, rate 5* | 1 | 0 | 0 | 1 | 667 | 164 | 167 | 331 |
|  | *0.25x, rate 5* | 347 | 3 | 151 | 38 | 110 | 218 | 133 | 351 |
|  | *0.5x, rate 5* | 64 | 0 | 40 | 2 | 6 | 745 | 143 | 888 |
|  | *0.75x, rate 5* | 0 | 0 | 0 | 0 | 2 | 883 | 115 | 998 |
|  | *1x, rate 5* | 0 | 0 | 0 | 0 | 0 | 921 | 79 | 1000 |
| ***Tree 200*** | *0.05x, rate 10* | 0 | 0 | 0 | 0 | 771 | 72 | 157 | 229 |
|  | *0.25x, rate 10* | 83 | 1 | 91 | 30 | 411 | 180 | 204 | 384 |
|  | *0.5x, rate 10* | 127 | 0 | 158 | 14 | 26 | 456 | 219 | 675 |
|  | *0.75x, rate 10* | 12 | 0 | 36 | 0 | 1 | 712 | 239 | 951 |
|  | *1x, rate 10* | 0 | 0 | 1 | 0 | 0 | 821 | 178 | 999 |
| ***Tree 500*** | *0.05x, rate 2* | 391 | 7 | 50 | 42 | 235 | 81 | 194 | 275 |
|  | *0.25x, rate 2* | 0 | 0 | 0 | 0 | 10 | 884 | 106 | 990 |
|  | *0.5x, rate 2* | 0 | 0 | 0 | 0 | 0 | 948 | 52 | 1000 |
|  | *0.75x, rate 2* | 0 | 0 | 0 | 0 | 0 | 963 | 37 | 1000 |
|  | *1x, rate 2* | 0 | 0 | 0 | 0 | 0 | 971 | 29 | 1000 |
| ***Tree 500*** | *0.05x, rate 5* | 0 | 0 | 0 | 0 | 814 | 0 | 186 | 186 |
|  | *0.25x, rate 5* | 155 | 1 | 213 | 10 | 9 | 275 | 337 | 612 |
|  | *0.5x, rate 5* | 0 | 0 | 0 | 0 | 0 | 771 | 229 | 1000 |
|  | *0.75x, rate 5* | 0 | 0 | 0 | 0 | 0 | 897 | 103 | 1000 |
|  | *1x, rate 5* | 0 | 0 | 0 | 0 | 0 | 942 | 58 | 1000 |
| ***Tree 500*** | *0.05x, rate 10* | 0 | 0 | 0 | 0 | 872 | 0 | 128 | 128 |
|  | *0.25x, rate 10* | 25 | 0 | 326 | 6 | 117 | 20 | 506 | 526 |
|  | *0.5x, rate 10* | 0 | 0 | 0 | 0 | 0 | 440 | 560 | 1000 |
|  | *0.75x, rate 10* | 0 | 0 | 0 | 0 | 0 | 725 | 275 | 1000 |
|  | *1x, rate 10* | 0 | 0 | 0 | 0 | 0 | 851 | 149 | 1000 |

**Supplementary Table S2.** The support for models with data simulated under the *nested EB rate* model. Results are shown for different tree sizes, and for several different rates of the EB rate parameter (*r*) and different shift rates. Different values of *r* were based on transformations of the maximum value used in simulations (1x, 0.95x, 0.75x, 0.5x, 0.25x, and 0.05x the maximum rate -9.21), and shifts are based on 2, 5, 10x the background BM rate. Support for the correct nested EB model increases with tree size, and support for one form of the nested EB model increases to over 95% on trees with 100 tips at the higher parameter values.


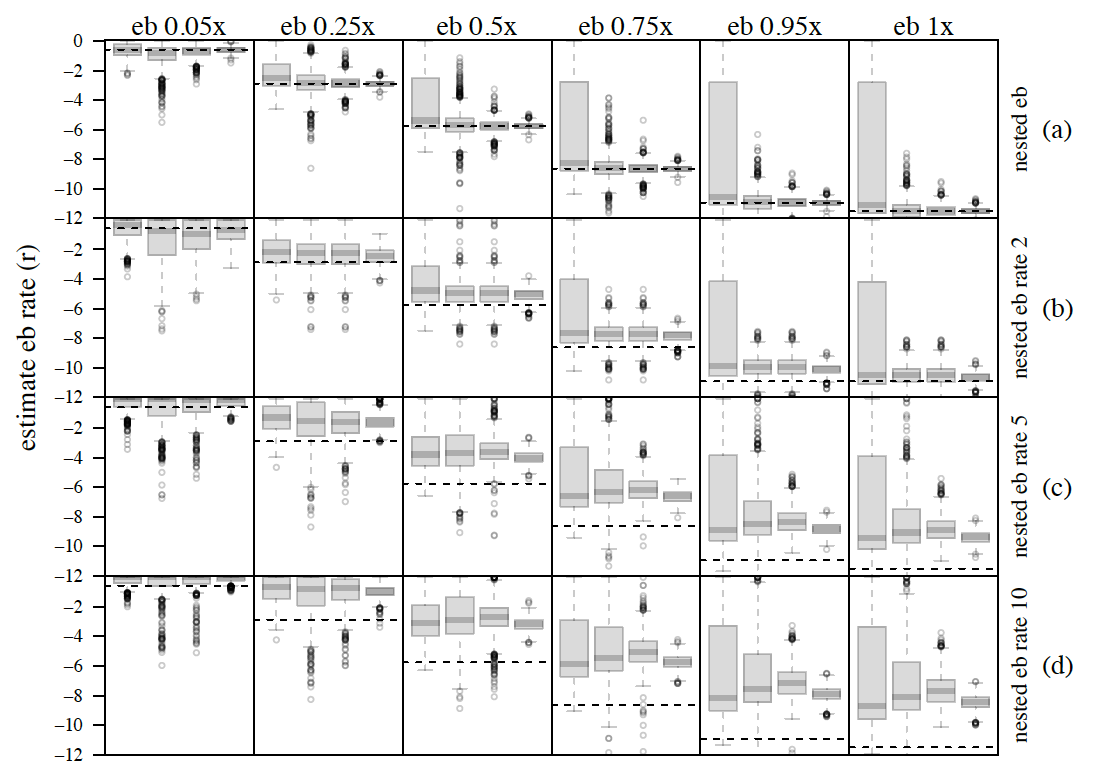


**Supplementary Figure S2**. The estimates of the early burst parameter *r* for all trees compared to the true rate (dotted black line) for the *nested EB* and *nested EB rate* models when data are simulated under each of these model respectively. For the *nested EB* model (a) the rate in the nested clade is equal to the ancestral rate (θ effectively is equal to 1). With the *nested EB rate* models the scalar value θ is set to 2x (b), 5x (c), and 10x (d) the background rate. In each box the data are shown for trees with 50, 100, 200, and 500 tips in order. For the *nested EB* model there is good accuracy and precision of the estimates, and precision increases with tree size. However, for the *nested EB rate* model the estimated value of *r* is lower than the true value.

|  |  |  | *BM* | *OU* | *EB* | *nested OU* | *nested shift* | *nested EB* | *nested EB rate* | Both *nested EB* model |
| --- | --- | --- | --- | --- | --- | --- | --- | --- | --- | --- |
| *EB* | Tree 50 | *0.05x* | 785 | 22 | 74 | 23 | 29 | 51 | 16 | 67 |
|  |  | *0.25x* | 246 | 0 | 697 | 2 | 14 | 38 | 3 | 41 |
|  |  | *0.5x* | 1 | 0 | 997 | 0 | 0 | 2 | 0 | 2 |
|  |  | *0.75x* | 0 | 0 | 1000 | 0 | 0 | 0 | 0 | 0 |
|  |  | *0.95x* | 0 | 0 | 1000 | 0 | 0 | 0 | 0 | 0 |
|  |  | *1x* | 0 | 0 | 1000 | 0 | 0 | 0 | 0 | 0 |
|  | Tree 100 | *0.05x* | 793 | 18 | 87 | 31 | 15 | 33 | 23 | 56 |
|  |  | *0.25x* | 123 | 0 | 828 | 3 | 5 | 26 | 15 | 41 |
|  |  | *0.5x* | 1 | 0 | 999 | 0 | 0 | 0 | 0 | 0 |
|  |  | *0.75x* | 0 | 0 | 1000 | 0 | 0 | 0 | 0 | 0 |
|  |  | *0.95x* | 0 | 0 | 1000 | 0 | 0 | 0 | 0 | 0 |
|  |  | *1x* | 0 | 0 | 1000 | 0 | 0 | 0 | 0 | 0 |
|  | Tree 200 | *0.05x* | 754 | 18 | 116 | 19 | 20 | 39 | 34 | 73 |
|  |  | *0.25x* | 72 | 0 | 895 | 1 | 4 | 13 | 15 | 28 |
|  |  | *0.5x* | 1 | 0 | 999 | 0 | 0 | 0 | 0 | 0 |
|  |  | *0.75x* | 0 | 0 | 1000 | 0 | 0 | 0 | 0 | 0 |
|  |  | *0.95x* | 0 | 0 | 1000 | 0 | 0 | 0 | 0 | 0 |
|  |  | *1x* | 0 | 0 | 1000 | 0 | 0 | 0 | 0 | 0 |
|  | Tree 500 | *0.05x* | 636 | 8 | 245 | 6 | 23 | 46 | 36 | 82 |
|  |  | *0.25x* | 2 | 0 | 995 | 0 | 0 | 0 | 3 | 3 |
|  |  | *0.5x* | 0 | 0 | 1000 | 0 | 0 | 0 | 0 | 0 |
|  |  | *0.75x* | 0 | 0 | 1000 | 0 | 0 | 0 | 0 | 0 |
|  |  | *0.95x* | 0 | 0 | 1000 | 0 | 0 | 0 | 0 | 0 |
|  |  | *1x* | 0 | 0 | 1000 | 0 | 0 | 0 | 0 | 0 |
| *OU* | Tree 50 | *0.05x* | 535 | 299 | 0 | 102 | 17 | 24 | 23 | 47 |
|  |  | *0.25x* | 247 | 600 | 0 | 97 | 14 | 14 | 28 | 42 |
|  |  | *0.5x* | 13 | 919 | 0 | 45 | 0 | 0 | 23 | 23 |
|  |  | *0.75x* | 0 | 975 | 0 | 7 | 0 | 0 | 18 | 18 |
|  |  | *0.95x* | 0 | 975 | 0 | 2 | 0 | 0 | 23 | 23 |
|  |  | *1x* | 0 | 971 | 0 | 1 | 0 | 0 | 28 | 28 |
|  | Tree 100 | *0.05x* | 411 | 481 | 0 | 67 | 21 | 14 | 6 | 20 |
|  |  | *0.25x* | 87 | 841 | 0 | 46 | 15 | 7 | 4 | 11 |
|  |  | *0.5x* | 1 | 991 | 0 | 5 | 2 | 0 | 1 | 1 |
|  |  | *0.75x* | 0 | 998 | 0 | 0 | 1 | 0 | 1 | 1 |
|  |  | *0.95x* | 0 | 999 | 0 | 0 | 0 | 0 | 1 | 1 |
|  |  | *1x* | 0 | 999 | 0 | 0 | 0 | 0 | 1 | 1 |
|  | Tree 200 | *0.05x* | 308 | 580 | 0 | 85 | 17 | 7 | 3 | 10 |
|  |  | *0.25x* | 29 | 934 | 0 | 31 | 4 | 2 | 0 | 2 |
|  |  | *0.5x* | 0 | 1000 | 0 | 0 | 0 | 0 | 0 | 0 |
|  |  | *0.75x* | 0 | 1000 | 0 | 0 | 0 | 0 | 0 | 0 |
|  |  | *0.95x* | 0 | 1000 | 0 | 0 | 0 | 0 | 0 | 0 |
|  |  | *1x* | 0 | 1000 | 0 | 0 | 0 | 0 | 0 | 0 |
|  | Tree 500 | *0.05x* | 16 | 924 | 0 | 57 | 1 | 1 | 1 | 2 |
|  |  | *0.25x* | 0 | 985 | 0 | 15 | 0 | 0 | 0 | 0 |
|  |  | *0.5x* | 0 | 999 | 0 | 1 | 0 | 0 | 0 | 0 |
|  |  | *0.75x* | 0 | 1000 | 0 | 0 | 0 | 0 | 0 | 0 |
|  |  | *0.95x* | 0 | 1000 | 0 | 0 | 0 | 0 | 0 | 0 |
|  |  | *1x* | 0 | 1000 | 0 | 0 | 0 | 0 | 0 | 0 |
| *Nested OU* | Tree 50 | *0.05x* | 658 | 95 | 13 | 143 | 17 | 55 | 19 | 74 |
|  |  | *0.25x* | 507 | 127 | 12 | 264 | 15 | 53 | 22 | 75 |
|  |  | *0.5x* | 313 | 130 | 6 | 461 | 19 | 46 | 25 | 71 |
|  |  | *0.75x* | 194 | 90 | 6 | 588 | 34 | 44 | 44 | 88 |
|  |  | *0.95x* | 158 | 66 | 5 | 573 | 57 | 48 | 93 | 141 |
|  |  | *1x* | 152 | 62 | 4 | 545 | 64 | 54 | 119 | 173 |
|  | Tree 100 | *0.05x* | 736 | 80 | 14 | 88 | 25 | 35 | 22 | 57 |
|  |  | *0.25x* | 617 | 92 | 14 | 178 | 34 | 41 | 24 | 65 |
|  |  | *0.5x* | 372 | 71 | 7 | 393 | 81 | 38 | 38 | 76 |
|  |  | *0.75x* | 116 | 37 | 6 | 547 | 214 | 22 | 58 | 80 |
|  |  | *0.95x* | 23 | 18 | 3 | 458 | 358 | 12 | 128 | 140 |
|  |  | *1x* | 12 | 15 | 4 | 408 | 404 | 9 | 148 | 157 |
|  | Tree 200 | *0.05x* | 668 | 87 | 9 | 142 | 36 | 36 | 22 | 58 |
|  |  | *0.25x* | 430 | 91 | 6 | 371 | 52 | 21 | 29 | 50 |
|  |  | *0.5x* | 77 | 43 | 2 | 781 | 60 | 7 | 30 | 37 |
|  |  | *0.75x* | 4 | 14 | 0 | 839 | 80 | 2 | 61 | 63 |
|  |  | *0.95x* | 1 | 4 | 0 | 613 | 172 | 0 | 210 | 210 |
|  |  | *1x* | 0 | 3 | 0 | 506 | 200 | 0 | 291 | 291 |
|  | Tree 500 | *0.05x* | 362 | 144 | 3 | 408 | 25 | 35 | 23 | 58 |
|  |  | *0.25x* | 95 | 68 | 2 | 798 | 13 | 13 | 11 | 24 |
|  |  | *0.5x* | 1 | 3 | 0 | 988 | 5 | 1 | 2 | 3 |
|  |  | *0.75x* | 0 | 0 | 0 | 968 | 15 | 0 | 17 | 17 |
|  |  | *0.95x* | 0 | 0 | 0 | 708 | 83 | 0 | 209 | 209 |
|  |  | *1x* | 0 | 0 | 0 | 548 | 95 | 0 | 357 | 357 |
| *Nested shift* | Tree 50 | 2x | 712 | 40 | 18 | 13 | 165 | 3 | 49 | 52 |
|  |  | 5x | 176 | 21 | 5 | 1 | 620 | 0 | 177 | 177 |
|  |  | 10x | 19 | 6 | 2 | 0 | 769 | 0 | 204 | 204 |
|  | Tree 100 | 2x | 370 | 46 | 10 | 49 | 352 | 93 | 80 | 173 |
|  |  | 5x | 1 | 0 | 0 | 3 | 794 | 55 | 147 | 202 |
|  |  | 10x | 0 | 0 | 0 | 0 | 836 | 27 | 137 | 164 |
|  | Tree 200 | 2x | 112 | 18 | 5 | 39 | 527 | 189 | 110 | 299 |
|  |  | 5x | 0 | 0 | 0 | 0 | 788 | 59 | 153 | 212 |
|  |  | 10x | 0 | 0 | 0 | 0 | 816 | 24 | 160 | 184 |
|  | Tree 500 | 2x | 9 | 0 | 1 | 4 | 890 | 7 | 89 | 96 |
|  |  | 5x | 0 | 0 | 0 | 0 | 893 | 0 | 107 | 107 |
|  |  | 10x | 0 | 0 | 0 | 0 | 889 | 0 | 111 | 111 |

**Supplementary Table S3.** The support for models with data simulated under the *EB, OU, nested OU,* and *rate shift* models. There is low erroneous support for the two *nested EB* models, but there is higher erroneous support for *nested EB rate* with data simulated under the *nested OU* and *nested shift* models.


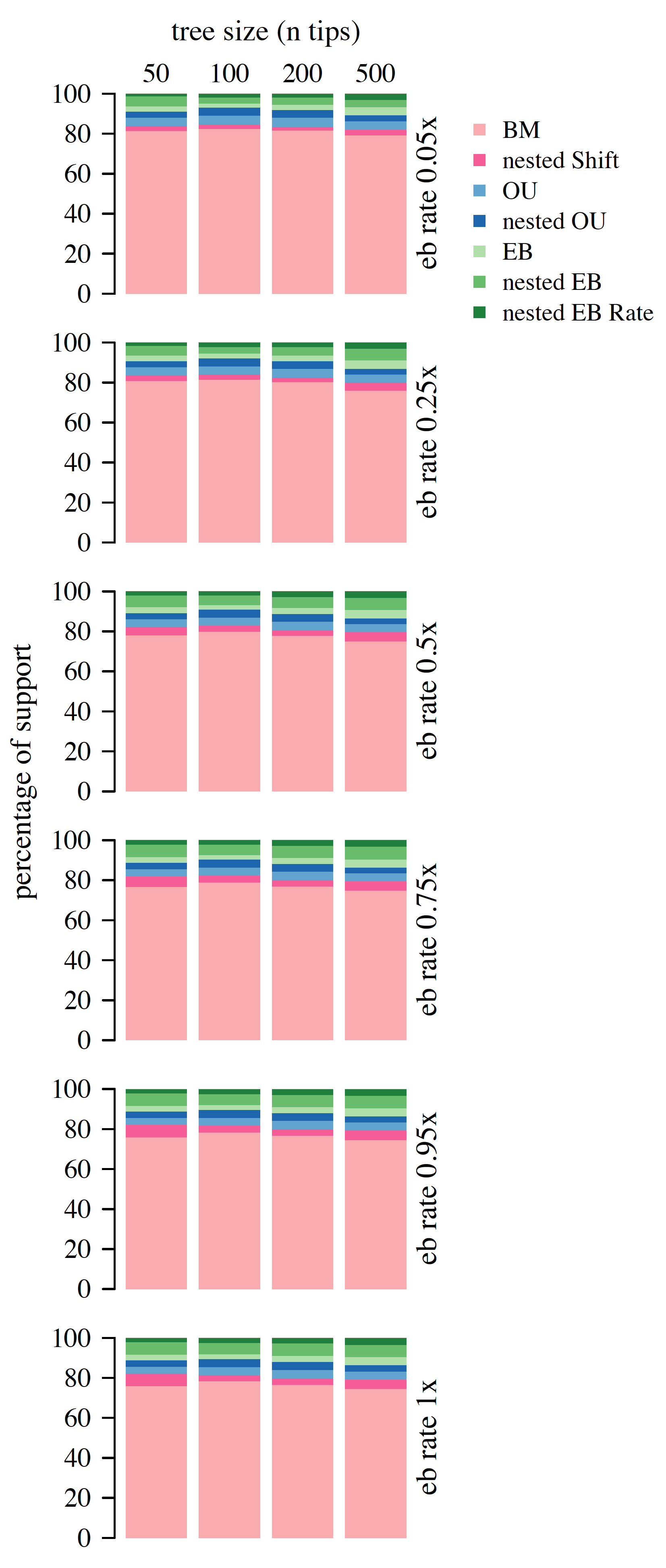


**Supplementary Figure S3**. **Simulation results showing the relative support for each model (as judged by AICc values) when data are simulated under the *nested EB* model, but shifts are only allowed in nodes ancestral to 5% of tips on the phylogeny and below.** However, when models are fit to these data shifts are only allowed on nodes that are ancestral to 25% of the phylogeny and above. In these cases the *nested EB* models are not able to detect these EB processes. With each model the results are summarised when data were simulated with different values of the EB parameter *r* (0.05, 0.25, 0.5, 0.75, and 1x the maximum rate). Generally the BM model receives the most support.


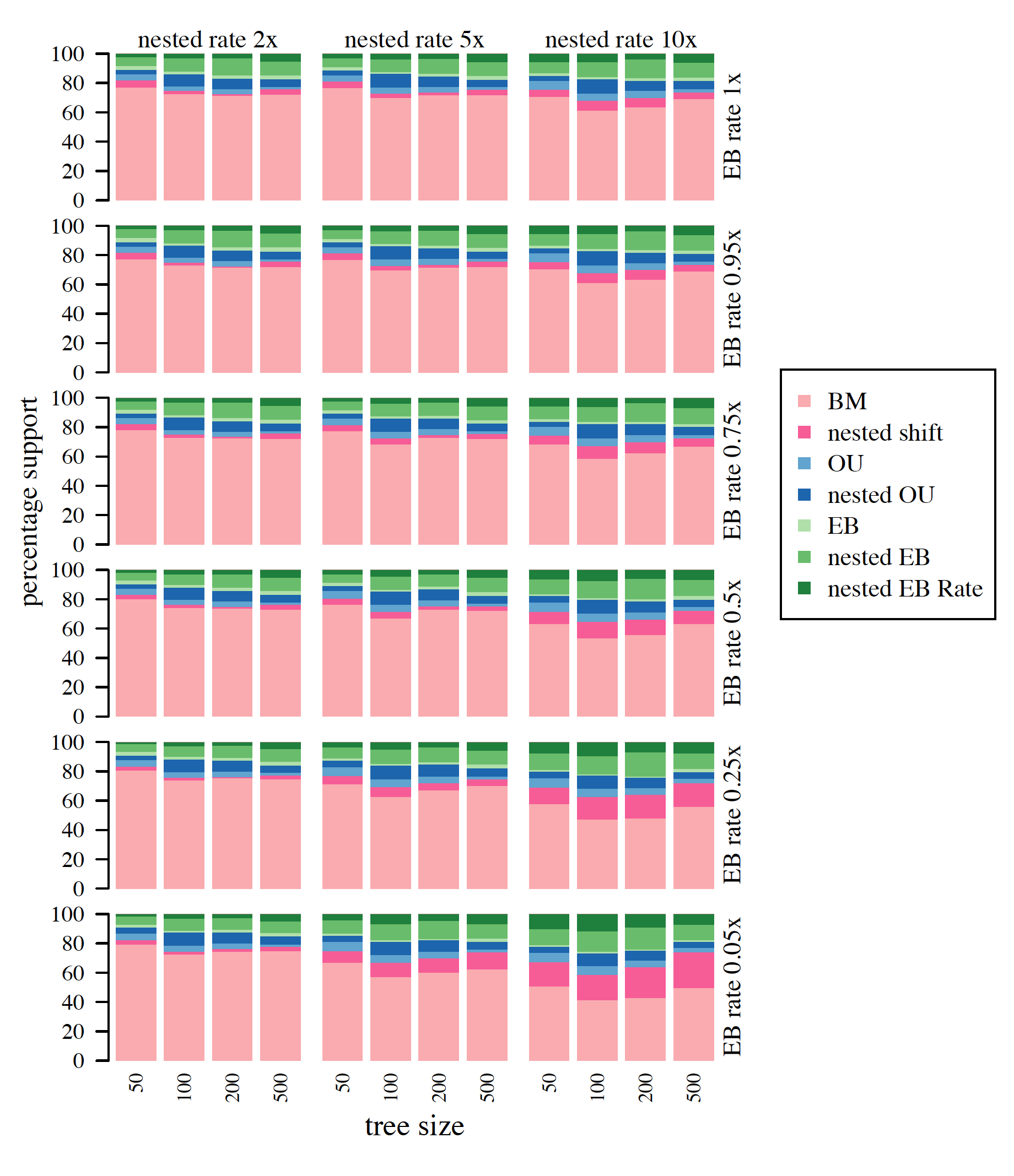


**Supplementary Figure S4**. Simulation results showing the relative support for each model (as judged by AICc values) when data are simulated under the *nested EB rate* model, but shifts are only allowed in nodes ancestral to 5% of tips on the phylogeny and below. However, in the model shifts are only allowed on nodes that are ancestral to 25% of the phylogeny and above. With each model the results are summarised when data were simulated with different values of the EB parameter *r* (0.05, 0.25, 0.5, 0.75, and 1x the maximum rate), and with different shift rates (2x, 5x, 10x the background rate). Generally the BM model receives the most support.

|  | model | log Likelihood | AICc | root state | *σ^2^* | eb rate (*r*) | shift scalar (θ) | Alpha (α) |
| --- | --- | --- | --- | --- | --- | --- | --- | --- |
| Artiodactyla | BM | -211.18 | 0.0016802 | 11.238 | 0.048096 | - | - | - |
|  | EB | -210.54 | 0.0011397 | 11.299 | 0.1299 | -0.01516 | - | - |
|  | OU | -211.18 | 0.00026173 | 11.238 | 0.048096 | - | - | 1.16E-08 |
|  | **nested EB rate** | **-199.94** | **0.99324** | **11.285** | **0.055952** | **-0.081197** | **16.995** | **-** |
|  | nested EB | -208.03 | 0.0034641 | 11.234 | 0.055687 | -0.031218 | - | - |
|  | nested OU | -211.18 | 0.00011925 | 11.238 | 0.048096 | - | - | 1.00E-08 |
|  | nested Shift | -209.74 | 9.47e-05 | 11.24 | 0.053513 | - | 0.6712 | - |
|  |  |  |  |  |  |  |  |  |
| Bovidae | BM | -129.28 | 0.00049226 | 10.663 | 0.046346 | - | - | - |
|  | EB | -127.47 | 0.0010522 | 10.638 | 0.10357 | -0.036578 | - | - |
|  | OU | -129.28 | 7.38e-05 | 10.663 | 0.046346 | - | - | 1.02E-08 |
|  | **nested EB rate** | **-117.87** | **0.99719** | **11.139** | **0.064818** | **-0.086132** | **16.13** | - |
|  | nested EB | -126 | 0.0010709 | 10.623 | 0.060374 | -0.035363 | - | - |
|  | nested OU | -129.28 | 3.01e-05 | 10.663 | 0.046346 | - | - | 1.00E-08 |
|  | nested Shift | -127.87 | 8.94e-05 | 10.398 | 0.071264 | - | 0.56629 | - |
|  |  |  |  |  |  |  |  |  |
| Carnivora | BM | -286.49 | 0.017087 | 9.4126 | 0.054651 | - | - | - |
|  | EB | -286.22 | 0.0080061 | 9.4146 | 0.086812 | -0.0077898 | - | - |
|  | OU | -286.49 | 0.0033057 | 9.4126 | 0.054651 | - | - | 1.00E-08 |
|  | **nested EB rate** | **-279.66** | **0.37096** | **9.693** | **0.062721** | **-0.056878** | **8.4243** | - |
|  | nested EB | -281.11 | 0.29305 | 9.3896 | 0.063297 | -0.02338 | - | - |
|  | nested OU | -282.67 | 0.044885 | 9.0072 | 0.061367 | - | - | 0.034925 |
|  | nested Shift | -280.69 | 0.2627 | 9.2205 | 0.064516 | - | 0.46484 | - |
|  |  |  |  |  |  |  |  |  |
| Chiroptera | BM | -620.28 | 6.92e-11 | 2.7884 | 0.022302 | - | - | - |
|  | EB | -620.28 | 2.03e-11 | 2.7884 | 0.022303 | -1.00E-06 | - | - |
|  | OU | -620.28 | 2.53e-11 | 2.7884 | 0.022302 | - | - | 1.00E-08 |
|  | **nested EB rate** | **-593.42** | **0.99999** | **3.2793** | **0.030461** | **-0.029598** | **16.305** | - |
|  | nested EB | -609.64 | 3.27e-07 | 2.7688 | 0.02638 | -0.0088065 | - | - |
|  | nested OU | -613.32 | 2.64e-08 | 2.7407 | 0.0243 | - | - | 0.027837 |
|  | nested Shift | -607.87 | 8.55e-07 | 2.7436 | 0.026864 | - | 0.58357 | - |
|  |  |  |  |  |  |  |  |  |
| Cricetidae | BM | -241.16 | 8.93e-07 | 4.3529 | 0.015154 | - | - | - |
|  | **EB** | **-226.32** | **0.90398** | **4.5472** | **0.33327** | **-0.072587** | - | - |
|  | OU | -241.16 | 1.75e-07 | 4.3529 | 0.015154 | - | - | 1.00E-08 |
|  | nested EB rate | -226.55 | 0.090513 | 4.4178 | 0.025006 | -0.044836 | 3.5287 | - |
|  | nested EB | -230.46 | 0.0054768 | 4.3557 | 0.026231 | -0.021699 | - | - |
|  | nested OU | -240.51 | 2.61e-07 | 4.3526 | 0.015618 | - | - | 0.014611 |
|  | nested Shift | -235.41 | 2.36e-05 | 4.3046 | 0.022637 | - | 0.585 | - |
|  |  |  |  |  |  |  |  |  |
| Diprotodontia | BM | -124.4 | 2.91e-07 | 7.1824 | 0.043534 | - | - | - |
|  | EB | -120.62 | 4.40e-06 | 7.3239 | 0.20174 | -0.035681 | - | - |
|  | OU | -124.4 | 3.68e-08 | 7.1824 | 0.043534 | - | - | 1.00E-08 |
|  | **nested EB rate** | **-105.25** | **0.99999** | **7.9749** | **0.099165** | **-0.061518** | **20** | - |
|  | nested EB | -121.97 | 3.32e-07 | 7.1171 | 0.1089 | -0.026888 | - | - |
|  | nested OU | -124.12 | 1.72e-08 | 7.2712 | 0.046759 | - | - | 0.011563 |
|  | nested Shift | -123.1 | 3.75e-08 | 7.1816 | 0.036312 | - | 1.6034 | - |
|  |  |  |  |  |  |  |  |  |
| Muridae | BM | -393.49 | 1.20e-08 | 4.0471 | 0.025352 | - | - | - |
|  | EB | -391.59 | 2.58e-08 | 4.0389 | 0.056569 | -0.019844 | - | - |
|  | OU | -393.49 | 1.83e-09 | 4.0471 | 0.025352 | - | - | 1.00E-08 |
|  | **nested EB rate** | **-371.13** | **0.99999** | **3.9333** | **0.026283** | **-0.050686** | **20** | - |
|  | nested EB | -387.95 | 2.36e-07 | 4.0437 | 0.028876 | -0.023697 | - | - |
|  | nested OU | -393.49 | 4.36e-09 | 4.0471 | 0.025352 | - | - | 1.00E-08 |
|  | nested Shift | -388.06 | 1.05e-07 | 4.0394 | 0.021598 | - | 1.667 | - |
|  |  |  |  |  |  |  |  |  |
| Phyllostomidae | BM | -100.45 | 0.065189 | 2.9896 | 0.023497 | - | - | - |
|  | EB | -100.45 | 0.022783 | 2.9896 | 0.023498 | -1.00E-06 | - | - |
|  | OU | -100.34 | 0.0098519 | 2.9827 | 0.025468 | - | - | 0.0062642 |
|  | nested EB rate | -94.709 | 0.32445 | 2.985 | 0.014028 | -1.00E-06 | 2.3996 | - |
|  | nested EB | -100.45 | 0.0071157 | 2.9896 | 0.023498 | -1.00E-06 | - | - |
|  | nested OU | -99.801 | 0.0070824 | 2.9137 | 0.028142 | - | - | 0.022431 |
|  | **nested Shift** | **-94.709** | **0.56353** | **2.985** | **0.014028** | **-** | **2.3996** | - |
|  |  |  |  |  |  |  |  |  |
| Primates | BM | -102.48 | 3.16e-08 | 6.816 | 0.014176 | - | - | - |
|  | **EB** | **-84.179** | **0.99957** | **6.6079** | **1.7676** | **-0.062579** | - | - |
|  | OU | -102.48 | 3.46e-09 | 6.816 | 0.014176 | - | - | 1.00E-08 |
|  | nested EB rate | -89.157 | 0.00041929 | 6.9114 | 0.016346 | -0.085183 | 4.5115 | - |
|  | nested EB | -95.237 | 6.23e-06 | 6.8682 | 0.018052 | -0.042894 | - | - |
|  | nested OU | -102.47 | 1.88e-09 | 6.8146 | 0.014279 | - | - | 0.0023762 |
|  | nested Shift | -101.34 | 5.76e-09 | 6.7882 | 0.015772 | - | 0.73264 | - |
|  |  |  |  |  |  |  |  |  |
| Pteropodidae | BM | -111.96 | 0.016609 | 4.1353 | 0.032016 | - | - | - |
|  | EB | -111.6 | 0.0083566 | 4.1272 | 0.046745 | -0.017855 | - | - |
|  | OU | -111.96 | 0.0024938 | 4.1353 | 0.032016 | - | - | 1.00E-08 |
|  | **nested EB rate** | **-104.25** | **0.93925** | **3.6103** | **0.018569** | **-0.043079** | **18.808** | - |
|  | nested EB | -109.27 | 0.022528 | 4.0702 | 0.036739 | -0.033454 | - | - |
|  | nested OU | -111.22 | 0.0020577 | 3.7486 | 0.035556 | - | - | 0.038528 |
|  | nested Shift | -109.59 | 0.008708 | 3.9264 | 0.037053 | - | 0.49749 | - |
|  |  |  |  |  |  |  |  |  |
| Rodentia | BM | -1310.8 | 5.39e-23 | 5.1481 | 0.026036 | - | - | - |
|  | EB | -1296 | 5.54e-17 | 5.2399 | 0.17572 | -0.02505 | - | - |
|  | OU | -1310.8 | 1.98e-23 | 5.1481 | 0.026036 | - | - | 1.00E-08 |
|  | **nested EB rate** | **-1255.7** | **0.92856** | **5.1525** | **0.031407** | **-0.040421** | **3.4719** | - |
|  | nested EB | -1259.6 | 0.071381 | 5.1496 | 0.031466 | -0.021018 | - | - |
|  | nested OU | -1310.8 | 1.98e-23 | 5.1481 | 0.026036 | - | - | 1.00E-08 |
|  | nested Shift | -1266.2 | 6.06e-05 | 5.1481 | 0.031085 | - | 0.43343 | - |
|  |  |  |  |  |  |  |  |  |
| Sciuridae | BM | -243.53 | 0.018309 | 5.0397 | 0.042324 | - | - | - |
|  | EB | -243.53 | 0.0050349 | 5.0397 | 0.042326 | -1.00E-06 | - | - |
|  | OU | -243.53 | 0.0055448 | 5.0397 | 0.042324 | - | - | 1.00E-08 |
|  | nested EB rate | -236.49 | 0.30218 | 5.0306 | 0.0321 | -0.012733 | 3.0346 | - |
|  | nested EB | -242.87 | 0.0020456 | 5.0472 | 0.046762 | -0.0060088 | - | - |
|  | nested OU | -243.53 | 0.00094757 | 5.0397 | 0.042324 | - | - | 1.00E-08 |
|  | **nested Shift** | **-236.7** | **0.66593** | **5.0354** | **0.031936** | **-** | **2.0255** | - |
|  |  |  |  |  |  |  |  |  |
| Soricidae | BM | -179.31 | 6.25E-08 | 2.4997 | 0.025503 | - | - | - |
|  | EB | -179.31 | 2.22E-08 | 2.4997 | 0.025504 | -1.00E-06 | - | - |
|  | OU | -177.5 | 7.13E-08 | 2.4814 | 0.035629 | - | - | 0.02356 |
|  | nested EB rate | -160.68 | 0.25021 | 2.4996 | 0.03516 | -0.055339 | 2.6381 | - |
|  | **nested EB** | **-160.93** | **0.50271** | **2.496** | **0.035287** | **-0.039644** | - | - |
|  | nested OU | -171.75 | 6.91E-06 | 2.1789 | 0.029296 | - | - | 0.047169 |
|  | nested Shift | -161.32 | 0.24708 | 2.4937 | 0.0088856 | - | 4.0248 | - |
|  |  |  |  |  |  |  |  |  |
| Soricomorpha | BM | -216 | 5.28e-08 | 3.943 | 0.02379 | - | - | - |
|  | EB | -216 | 1.89e-08 | 3.943 | 0.023792 | -1.00E-06 | - | - |
|  | OU | -215.96 | 1.26e-08 | 3.922 | 0.024451 | - | - | 0.0014544 |
|  | nested EB rate | -196.53 | 0.35643 | 3.9638 | 0.010258 | -1.00E-06 | 3.4912 | - |
|  | nested EB | -200.03 | 0.038384 | 3.9304 | 0.030784 | -0.035835 | - | - |
|  | nested OU | -210.16 | 8.20e-07 | 3.7794 | 0.026664 | - | - | 0.041842 |
|  | **nested Shift** | **-196.53** | **0.60518** | **3.9638** | **0.010258** | **-** | **3.4911** | - |
|  |  |  |  |  |  |  |  |  |
| Vespertilionidae | BM | -143.29 | 0.24833 | 2.2852 | 0.016855 | - | - | - |
|  | EB | -143.29 | 0.088922 | 2.2852 | 0.016855 | -1.00E-06 | - | - |
|  | **OU** | **-140.98** | **0.34729** | **2.2701** | **0.022377** | **-** | **-** | **0.024349** |
|  | nested EB rate | -139.43 | 0.14873 | 2.292 | 0.011844 | -0.033222 | 5.9139 | - |
|  | nested EB | -143.29 | 0.01746 | 2.2852 | 0.016855 | -1.00E-06 | - | - |
|  | nested OU | -142.25 | 0.027945 | 2.2602 | 0.020295 | - | - | 0.016188 |
|  | nested Shift | -140.28 | 0.12132 | 2.2977 | 0.011824 | - | 1.648 | - |

**Supplementary Table S4.** A summary of model parameters for models fit to body size evolution in mammalian clades. The table summarises the log-likelihood, corrected AICc weights, σ^2^, root state, early burst parameter *r* (only applicable to *nested EB* and *nested EB rate*), scalar rate for shift clade (only applicable to *nested EB rate* and *nested shift*), and alpha value (only applicable to *OU* and *nested OU*). The model with the highest relative fit is highlighted in **bold**.

|  | model | log Likelihood | AICc | root state | *σ^2^* | eb rate (*r*) | shift scalar (θ) | Alpha (α) |
| --- | --- | --- | --- | --- | --- | --- | --- | --- |
| Accipitridae | BM | -126.73 | 0.041977 | 6.2242 | 0.032671 | - | - | - |
|  | EB | -126.73 | 0.014781 | 6.2242 | 0.032673 | -1.00E-06 | - | - |
|  | OU | -126.66 | 0.0072552 | 6.246 | 0.033983 | - | - | 0.0026901 |
|  | **nested EB rate** | **-119.35** | **0.91885** | **6.3397** | **0.022885** | **-0.04769** | **12.75** | - |
|  | nested EB | -126.73 | 0.0044294 | 6.2242 | 0.032673 | -1.00E-06 | - | - |
|  | nested OU | -126.73 | 0.0025224 | 6.2242 | 0.032671 | - | - | 1.00E-08 |
|  | nested Shift | -124.24 | 0.010181 | 6.2719 | 0.020017 | - | 1.8777 | - |
|  |  |  |  |  |  |  |  |  |
| Accipitriformes | BM | -141.15 | 0.032477 | 7.4262 | 0.031998 | - | - | - |
|  | EB | -141.15 | 0.011466 | 7.4262 | 0.032 | -1.00E-06 | - | - |
|  | OU | -140.91 | 0.0061233 | 7.3919 | 0.034275 | - | - | 0.0041307 |
|  | **nested EB rate** | **-133.21** | **0.93215** | **7.4687** | **0.02255** | **-0.047369** | **12.907** | - |
|  | nested EB | -141.15 | 0.0028414 | 7.4262 | 0.031998 | -1.00E-06 | - | - |
|  | nested OU | -141.15 | 0.0020024 | 7.4262 | 0.031998 | - | - | 1.00E-08 |
|  | nested Shift | -138.22 | 0.012938 | 7.4456 | 0.020011 | - | 1.8854 | - |
|  |  |  |  |  |  |  |  |  |
| Anatidae | BM | -75.612 | 0.068652 | 6.7994 | 0.035653 | - | - | - |
|  | EB | -75.612 | 0.024124 | 6.7994 | 0.035654 | -1.00E-06 | - | - |
|  | OU | -75.612 | 0.024125 | 6.7994 | 0.035653 | - | - | 1.00E-08 |
|  | nested EB rate | -69.585 | 0.38529 | 6.6487 | 0.0043764 | -0.0085467 | 12.678 | - |
|  | nested EB | -75.608 | 0.0061433 | 6.7994 | 0.035903 | -0.0020463 | - | - |
|  | nested OU | -74.345 | 0.011691 | 6.6733 | 0.041283 | - | - | 0.036054 |
|  | **nested Shift** | **-70.073** | **0.47998** | **6.6629** | **0.0044196** | **-** | **8.5721** | - |
|  |  |  |  |  |  |  |  |  |
| Anseriformes | BM | -80.263 | 0.030586 | 7.3936 | 0.035026 | - | - | - |
|  | EB | -80.263 | 0.010758 | 7.3936 | 0.035028 | -1.00E-06 | - | - |
|  | OU | -80.263 | 0.010759 | 7.3936 | 0.035026 | - | - | 1.00E-08 |
|  | **nested EB rate** | **-73.134** | **0.48082** | **7.3782** | **0.0051491** | **-0.0096906** | **11.049** | **-** |
|  | nested EB | -80.263 | 0.0025547 | 7.3936 | 0.035026 | -1.00E-06 | - | - |
|  | nested OU | -78.411 | 0.0094947 | 7.0595 | 0.041404 | - | - | 0.024398 |
|  | nested Shift | -73.554 | 0.45503 | 7.3852 | 0.0052014 | - | 7.28 | - |
|  | - | - | - | - | - | - | - | - |
| Apodiformes | BM | -71.853 | 0.00017702 | 3.0336 | 0.012225 | - | - | - |
|  | EB | -71.853 | 6.34e-05 | 3.0336 | 0.012226 | -1.00E-06 | - | - |
|  | OU | -71.853 | 6.34e-05 | 3.0336 | 0.012225 | - | - | 3.85E-08 |
|  | **nested EB rate** | **-59.03** | **0.98948** | **3.3747** | **0.015853** | **-0.075209** | **20** | - |
|  | nested EB | -65.825 | 0.0061691 | 3.0335 | 0.014446 | -0.044873 | - | - |
|  | nested OU | -68.156 | 0.00039961 | 2.2289 | 0.016289 | - | - | 0.041351 |
|  | nested Shift | -65.198 | 0.0036445 | 3.0333 | 0.0090953 | - | 1.9887 | - |
|  |  |  |  |  |  |  |  |  |
| Charadriiformes | BM | -186.26 | 1.46e-16 | 5.2345 | 0.031073 | - | - | - |
|  | EB | -186.26 | 5.23e-17 | 5.2345 | 0.031075 | -1.00E-06 | - | - |
|  | OU | -186.26 | 5.23e-17 | 5.2345 | 0.031073 | - | - | 1.00E-08 |
|  | **nested EB rate** | **-145.84** | **0.99832** | **5.1974** | **0.013099** | **-0.036491** | **20** | - |
|  | nested EB | -180.17 | 5.10e-15 | 5.2409 | 0.03716 | -0.016703 | - | - |
|  | nested OU | -179.05 | 1.22e-14 | 4.9151 | 0.038547 | - | - | 0.028024 |
|  | nested Shift | -152.92 | 0.0016835 | 5.1991 | 0.012976 | - | 5.0113 | - |
|  |  |  |  |  |  |  |  |  |
| Columbidae | BM | -77.614 | 0.023138 | 5.134 | 0.014075 | - | - | - |
|  | EB | -76.825 | 0.017826 | 5.1181 | 0.024745 | -0.018611 | - | - |
|  | OU | -77.614 | 0.0032113 | 5.134 | 0.014075 | - | - | 1.00E-08 |
|  | **nested EB rate** | **-70.197** | **0.79139** | **4.5707** | **0.014511** | **-0.053851** | **20** | - |
|  | nested EB | -76.831 | 0.0040511 | 5.1341 | 0.015389 | -0.018 | - | - |
|  | nested OU | -72.905 | 0.15749 | 5.1453 | 0.016685 | - | - | 0.049553 |
|  | nested Shift | -76.409 | 0.0028887 | 5.1419 | 0.015826 | - | 0.64535 | - |
|  |  |  |  |  |  |  |  |  |
| Cuculidae | **BM** | **-73.821** | **0.43028** | **4.8603** | **0.01276** | - | - | - |
|  | EB | -73.821 | 0.14897 | 4.8603 | 0.012761 | -1.00E-06 | - | - |
|  | OU | -73.279 | 0.12305 | 4.8418 | 0.014662 | - | - | 0.0064601 |
|  | nested EB rate | -71.449 | 0.096336 | 4.9037 | 0.012905 | -0.054508 | 14.883 | - |
|  | nested EB | -73.349 | 0.058664 | 4.8585 | 0.013689 | -0.0071772 | - | - |
|  | nested OU | -72.075 | 0.10228 | 4.8188 | 0.014132 | - | - | 0.027872 |
|  | nested Shift | -73.011 | 0.040421 | 4.8501 | 0.014049 | - | 0.66509 | - |
|  |  |  |  |  |  |  |  |  |
| Emberizidae | BM | -13.419 | 0.026739 | 3.2593 | 0.010234 | - | - | - |
|  | EB | -13.419 | 0.0092583 | 3.2593 | 0.010234 | -1.00E-06 | - | - |
|  | OU | -12.742 | 0.0065813 | 3.2071 | 0.012396 | - | - | 0.025907 |
|  | nested EB rate | -7.9331 | 0.099258 | 3.2476 | 0.0045567 | -1.00E-06 | 2.8563 | - |
|  | nested EB | -8.3716 | 0.20562 | 3.2547 | 0.012485 | -0.082947 | - | - |
|  | nested OU | -8.2303 | 0.18244 | 3.1451 | 0.012237 | - | - | 0.1267 |
|  | **nested Shift** | **-7.1568** | **0.4701** | **3.2006** | **0.013058** | **-** | **0.31218** | - |
|  |  |  |  |  |  |  |  |  |
| Furnariidae | **BM** | **-62.315** | **0.46827** | **3.2544** | **0.017644** | - | - | - |
|  | EB | -62.315 | 0.16671 | 3.2544 | 0.017645 | -1.00E-06 | - | - |
|  | OU | -62.315 | 0.16671 | 3.2544 | 0.017644 | - | - | 1.00E-08 |
|  | nested EB rate | -60.64 | 0.04233 | 3.256 | 0.014391 | -1.00E-06 | 1.4587 | - |
|  | nested EB | -62.218 | 0.044496 | 3.2543 | 0.018216 | -0.0090973 | - | - |
|  | nested OU | -62.233 | 0.026395 | 3.2524 | 0.018225 | - | - | 0.0073988 |
|  | nested Shift | -60.64 | 0.085082 | 3.256 | 0.014391 | - | 1.4587 | - |
|  |  |  |  |  |  |  |  |  |
| Galliformes | **BM** | **-98.876** | **0.3888** | **6.6849** | **0.025635** | - | - | - |
|  | EB | -98.598 | 0.18154 | 6.7013 | 0.0557 | -0.013927 | - | - |
|  | OU | -98.876 | 0.1375 | 6.6849 | 0.025635 | - | - | 1.00E-08 |
|  | nested EB rate | -96.595 | 0.065916 | 6.8485 | 0.0097521 | -0.0020792 | 3.1286 | - |
|  | nested EB | -98.706 | 0.052296 | 6.6932 | 0.028227 | -0.004813 | - | - |
|  | nested OU | -98.876 | 0.020519 | 6.6849 | 0.025635 | - | - | 1.81E-08 |
|  | nested Shift | -96.617 | 0.15343 | 6.8436 | 0.0094495 | - | 2.8561 | - |
|  |  |  |  |  |  |  |  |  |
| Muscicapidae | BM | -32.677 | 0.22976 | 2.9859 | 0.013404 | - | - | - |
|  | EB | -32.677 | 0.08105 | 2.9859 | 0.013405 | -1.00E-06 | - | - |
|  | OU | -32.677 | 0.041791 | 2.9859 | 0.013404 | - | - | 4.16E-07 |
|  | **nested EB rate** | **-28.533** | **0.32552** | **3.008** | **0.015844** | **-0.12669** | **5.9585** | - |
|  | nested EB | -30.417 | 0.17837 | 2.9859 | 0.019189 | -0.044958 | - | - |
|  | nested OU | -32.223 | 0.030066 | 2.9752 | 0.014769 | - | - | 0.017407 |
|  | nested Shift | -30.111 | 0.11345 | 2.9732 | 0.021208 | - | 0.53852 | - |
|  |  |  |  |  |  |  |  |  |
| Passeriformes | BM | -1261.6 | 3.13e-54 | 3.4005 | 0.014539 | - | - | - |
|  | EB | -1261.6 | 8.83e-55 | 3.4005 | 0.01454 | -1.00E-06 | - | - |
|  | OU | -1261.6 | 1.15e-54 | 3.4005 | 0.014539 | - | - | 1.00E-08 |
|  | **nested EB rate** | **-1134.6** | **0.99999** | **3.2139** | **0.055376** | **-0.048391** | **18.856** | - |
|  | nested EB | -1261.6 | 3.36e-55 | 3.4005 | 0.014539 | -1.00E-06 | - | - |
|  | nested OU | -1261.6 | 1.15e-54 | 3.4005 | 0.014539 | - | - | 1.00E-08 |
|  | nested Shift | -1247.2 | 3.03e-49 | 3.4008 | 0.013322 | - | 1.3501 | - |
|  |  |  |  |  |  |  |  |  |
| Picidae | BM | -54.785 | 0.15571 | 3.3326 | 0.022939 | - | - | - |
|  | EB | -53.181 | 0.27088 | 3.2695 | 0.071199 | -0.055937 | - | - |
|  | OU | -54.785 | 0.031953 | 3.3326 | 0.022939 | - | - | 1.00E-08 |
|  | **nested EB rate** | **-49.196** | **0.47493** | **2.9437** | **0.01341** | **-0.070755** | **8.3144** | - |
|  | nested EB | -53.399 | 0.041166 | 3.2401 | 0.0594 | -0.052206 | - | - |
|  | nested OU | -54.122 | 0.012955 | 3.3207 | 0.02472 | - | - | 0.033417 |
|  | nested Shift | -53.497 | 0.012396 | 3.5297 | 0.0011508 | - | 20 | - |
|  |  |  |  |  |  |  |  |  |
| Piciformes | BM | -105.26 | 0.062009 | 3.6777 | 0.021341 | - | - | - |
|  | EB | -104.92 | 0.031135 | 3.6733 | 0.04563 | -0.01506 | - | - |
|  | OU | -105.26 | 0.022172 | 3.6777 | 0.021341 | - | - | 1.00E-08 |
|  | **nested EB rate** | **-98.145** | **0.87082** | **3.573** | **0.013101** | **-0.043122** | **9.2945** | - |
|  | nested EB | -104.81 | 0.0060334 | 3.682 | 0.022751 | -0.0072376 | - | - |
|  | nested OU | -104.95 | 0.003291 | 3.6765 | 0.021976 | - | - | 0.021508 |
|  | nested Shift | -104.21 | 0.0045421 | 3.6099 | 0.010418 | - | 2.0996 | - |
|  |  |  |  |  |  |  |  |  |
| Procellariiformes | BM | -66.59 | 0.064099 | 4.9855 | 0.018348 | - | - | - |
|  | **EB** | **-63.097** | **0.72897** | **4.9407** | **0.090677** | **-0.0286** | - | - |
|  | OU | -66.59 | 0.006239 | 4.9855 | 0.018348 | - | - | 1.00E-08 |
|  | nested EB rate | -62.31 | 0.088688 | 4.9936 | 0.021794 | -0.068889 | 20 | - |
|  | nested EB | -64.187 | 0.062392 | 4.9782 | 0.02146 | -0.020662 | - | - |
|  | nested OU | -64.894 | 0.01611 | 4.9401 | 0.020042 | - | - | 0.041613 |
|  | nested Shift | -63.94 | 0.033505 | 4.963 | 0.021728 | - | 0.47003 | - |
|  |  |  |  |  |  |  |  |  |
| Psittacidae | BM | -134 | 0.0037409 | 4.6009 | 0.038578 | - | - | - |
|  | EB | -134 | 0.0012652 | 4.6022 | 0.040061 | -0.0017985 | - | - |
|  | OU | -134 | 0.0013336 | 4.6009 | 0.038578 | - | - | 1.00E-08 |
|  | nested EB rate | -126.62 | 0.1454 | 4.5828 | 0.045376 | -0.097089 | 2.476 | - |
|  | **nested EB** | **-126.64** | **0.53696** | **4.603** | **0.045533** | **-0.055829** | - | - |
|  | nested OU | -132.18 | 0.0010548 | 4.6591 | 0.040558 | - | - | 0.047576 |
|  | nested Shift | -126.76 | 0.31025 | 4.63 | 0.045744 | - | 0.38082 | - |
|  |  |  |  |  |  |  |  |  |
| Psittaciformes | BM | -154.77 | 0.034582 | 5.9999 | 0.039118 | - | - | - |
|  | EB | -154.77 | 0.011896 | 5.9999 | 0.03912 | -1.00E-06 | - | - |
|  | OU | -154.77 | 0.012362 | 5.9999 | 0.039118 | - | - | 1.00E-08 |
|  | nested EB rate | -148.12 | 0.34079 | 6.0022 | 0.049865 | -0.075246 | 3.1543 | - |
|  | **nested EB** | **-149.44** | **0.56441** | **5.9921** | **0.050682** | **-0.031662** | - | - |
|  | nested OU | -153.66 | 0.0053129 | 5.9585 | 0.042119 | - | - | 0.040455 |
|  | nested Shift | -151.08 | 0.030643 | 5.9863 | 0.048626 | - | 0.58782 | - |
|  |  |  |  |  |  |  |  |  |
| Strigiformes | BM | -76.326 | 0.1545 | 5.5522 | 0.019674 | - | - | - |
|  | EB | -76.326 | 0.053393 | 5.5522 | 0.019675 | -1.00E-06 | - | - |
|  | OU | -76.139 | 0.016391 | 5.5477 | 0.021193 | - | - | 0.0048825 |
|  | **nested EB rate** | **-71.486** | **0.37706** | **5.5505** | **0.031117** | **-0.059659** | **17.929** | - |
|  | nested EB | -73.759 | 0.27557 | 5.5492 | 0.027718 | -0.021435 | - | - |
|  | nested OU | -76.326 | 0.0081287 | 5.5522 | 0.019674 | - | - | 1.00E-08 |
|  | nested Shift | -73.107 | 0.11495 | 5.851 | 0.0028186 | - | 7.3742 | - |
|  |  |  |  |  |  |  |  |  |
| Thamnophilidae | BM | -27.621 | 1.38e-05 | 2.8214 | 0.0075383 | - | - | - |
|  | **EB** | **-15.425** | **0.96443** | **2.754** | **0.067994** | **-0.098513** | - | - |
|  | OU | -27.621 | 2.17e-06 | 2.8214 | 0.0075383 | - | - | 1.00E-08 |
|  | nested EB rate | -16.586 | 0.012627 | 2.8295 | 0.017273 | -0.094674 | 2.4084 | - |
|  | nested EB | -17.827 | 0.022921 | 2.9082 | 0.04144 | -0.086315 | - | - |
|  | nested OU | -27.621 | 8.49e-07 | 2.8214 | 0.0075383 | - | - | 1.00E-08 |
|  | nested Shift | -27.398 | 4.25e-07 | 2.8073 | 0.006495 | - | 1.2193 | - |
|  |  |  |  |  |  |  |  |  |
| Thraupidae | BM | -86.092 | 2.12e-06 | 3.1399 | 0.021789 | - | - | - |
|  | EB | -86.092 | 7.63e-07 | 3.1399 | 0.021789 | -1.00E-06 | - | - |
|  | OU | -86.092 | 7.63e-07 | 3.1399 | 0.021789 | - | - | 1.00E-08 |
|  | **nested EB rate** | **-68.456** | **0.99919** | **3.0847** | **0.016942** | **-0.11626** | **20** | - |
|  | nested EB | -82.102 | 8.04e-06 | 3.1341 | 0.026669 | -0.044076 | - | - |
|  | nested OU | -82.734 | 3.07e-06 | 3.1049 | 0.024832 | - | - | 0.054091 |
|  | nested Shift | -76.028 | 0.00079917 | 3.1463 | 0.016183 | - | 2.3127 | - |
|  |  |  |  |  |  |  |  |  |
| Trochilidae | BM | -44.325 | 2.06e-06 | 1.8813 | 0.011611 | - | - | - |
|  | EB | -44.325 | 7.37e-07 | 1.8813 | 0.011612 | -1.00E-06 | - | - |
|  | OU | -44.325 | 7.38e-07 | 1.8813 | 0.011611 | - | - | 1.00E-08 |
|  | **nested EB rate** | **-27.378** | **0.99788** | **1.9041** | **0.0079304** | **-0.075871** | **20** | - |
|  | nested EB | -39.033 | 4.80e-05 | 1.8813 | 0.01393 | -0.0427 | - | - |
|  | nested OU | -40.852 | 4.03e-06 | 1.7974 | 0.01475 | - | - | 0.036533 |
|  | nested Shift | -34.236 | 0.0020667 | 1.8789 | 0.0074796 | - | 2.4175 | - |
|  |  |  |  |  |  |  |  |  |
| Turdidae | BM | -0.0379 | 0.1609 | 3.9854 | 0.0096898 | - | - | - |
|  | EB | -0.036012 | 0.05601 | 3.9849 | 0.010025 | -0.0023228 | - | - |
|  | OU | -0.037903 | 0.019675 | 3.9854 | 0.0096899 | - | - | 9.07E-07 |
|  | nested EB rate | 2.1598 | 0.031383 | 3.7875 | 0.007526 | -0.089689 | 8.0748 | - |
|  | nested EB | -0.037903 | 0.017365 | 3.9854 | 0.0096899 | -1.00E-06 | - | - |
|  | **nested OU** | **3.8533** | **0.66775** | **4.0854** | **0.013694** | - | - | 0.14186 |
|  | nested Shift | 1.9026 | 0.046915 | 3.9856 | 0.0080435 | - | 1.7826 | - |
|  |  |  |  |  |  |  |  |  |
| Tyrannidae | BM | -92.302 | 2.00e-12 | 2.5294 | 0.015512 | - | - | - |
|  | EB | -92.302 | 7.18e-13 | 2.5294 | 0.015513 | -1.00E-06 | - | - |
|  | OU | -92.302 | 7.18e-13 | 2.5294 | 0.015512 | - | - | 1.00E-08 |
|  | **nested EB rate** | **-61.373** | **0.99997** | **2.4926** | **0.0076474** | **-0.058635** | **20** | - |
|  | nested EB | -81.458 | 8.28e-09 | 2.5258 | 0.018461 | -0.063036 | - | - |
|  | nested OU | -87.124 | 1.51e-11 | 2.5087 | 0.019498 | - | - | 0.031415 |
|  | nested Shift | -72.443 | 3.38e-05 | 2.5063 | 0.0076908 | - | 3.1362 | - |

**Supplementary Table S5.** A summary of model parameters for models fit to body size evolution in bird clades. The table summarises the log-likelihood, corrected AICc weights, σ^2^, root state, early burst parameter *r* (only applicable to *nested EB* and *nested EB rate*), scalar rate for shift clade (only applicable to *nested EB rate* and *nested shift*), and alpha value (only applicable to *OU* and *nested OU*). The model with the highest relative fit is highlighted in **bold**.

|  | model | log Likelihood | AICc | root state | *σ^2^* | eb rate (*r*) | shift scalar (θ) | Alpha (α) |
| --- | --- | --- | --- | --- | --- | --- | --- | --- |
| Agamidae | BM | -149.92 | 8.21e-05 | 3.8139 | 0.028543 | - | - | - |
|  | EB | -149.92 | 2.85e-05 | 3.8139 | 0.028546 | -1.00E-06 | - | - |
|  | OU | -149.66 | 1.31e-05 | 3.7512 | 0.031922 | - | - | 0.0035198 |
|  | nested EB rate | -137.35 | 0.27359 | 3.7882 | 0.015736 | -1.00E-06 | 4.1187 | - |
|  | nested EB | -140.1 | 0.072321 | 3.7518 | 0.051388 | -0.013717 | - | - |
|  | nested OU | -143.75 | 0.00184 | 3.5964 | 0.034543 | - | - | 0.066648 |
|  | **nested Shift** | **-137.35** | **0.65212** | **3.7882** | **0.015736** | **-** | **4.1185** | - |
|  |  |  |  |  |  |  |  |  |
| Colubridae | BM | -193.43 | 0.0043534 | 6.2985 | 0.073399 | - | - | - |
|  | EB | -192.47 | 0.0035366 | 6.2045 | 0.19778 | -0.02808 | - | - |
|  | OU | -193.43 | 0.00064502 | 6.2985 | 0.073399 | - | - | 1.46E-08 |
|  | nested EB rate | -184.75 | 0.38374 | 6.2984 | 0.0043831 | -1.00E-06 | 18.038 | - |
|  | nested EB | -193.16 | 0.00050367 | 6.2987 | 0.078044 | -0.010383 | - | - |
|  | nested OU | -193.35 | 0.00020116 | 6.3048 | 0.075365 | - | - | 0.0062216 |
|  | **nested Shift** | **-184.75** | **0.60702** | **6.2984** | **0.0043831** | **-** | **18.036** | - |
|  |  |  |  |  |  |  |  |  |
| Lacertidae | BM | -119.41 | 9.06e-05 | 2.0224 | 0.016997 | - | - | - |
|  | EB | -119.41 | 3.15e-05 | 2.0224 | 0.016998 | -1.00E-06 | - | - |
|  | OU | -119.41 | 1.25e-05 | 2.0224 | 0.016997 | - | - | 1.00E-08 |
|  | nested EB rate | -108.54 | 0.096364 | 2.1112 | 0.053699 | -0.040575 | 2.4803 | - |
|  | **nested EB** | **-108.39** | **0.53331** | **2.0473** | **0.052651** | **-0.028787** | - | - |
|  | nested OU | -119.41 | 6.84e-06 | 2.0224 | 0.016997 | - | - | 1.00E-08 |
|  | nested Shift | -107.8 | 0.37019 | 1.8792 | 0.081525 | - | 0.14831 | - |
|  |  |  |  |  |  |  |  |  |
| Scincidae | BM | -273.87 | 0.015986 | 2.0776 | 0.041446 | - | - | - |
|  | EB | -273.87 | 0.0056894 | 2.0776 | 0.04145 | -1.00E-06 | - | - |
|  | OU | -272.54 | 0.011278 | 2.1048 | 0.050844 | - | - | 0.0080942 |
|  | nested EB rate | -268.2 | 0.094323 | 2.059 | 0.052197 | -0.014192 | 1 | - |
|  | **nested EB** | **-268.2** | **0.68466** | **2.059** | **0.052197** | **-0.014192** | - | - |
|  | nested OU | -273.87 | 0.003166 | 2.0776 | 0.041446 | - | - | 4.07E-08 |
|  | nested Shift | -267.85 | 0.1849 | 2.0028 | 0.053303 | - | 0.47036 | - |

**Supplementary Table S6.** A summary of model parameters for models fit to body size evolution in squamate clades. The table summarises the log-likelihood, corrected AICc weights, σ^2^, root state, early burst parameter *r* (only applicable to *nested EB* and *nested EB rate*), scalar rate for shift clade (only applicable to *nested EB rate* and *nested shift*), and alpha value (only applicable to *OU* and *nested OU*). The model with the highest relative fit is highlighted in **bold**.

|  |  | whole phylogeny model | whole phylogeny aiccW | all models | all models aiccW |
| --- | --- | --- | --- | --- | --- |
| Mammalia Orders | Artiodactyla | BM | 0.49169 | nested EB Rate | 0.99872 |
|  | Carnivora | EB | 0.6132 | nested EB Rate | 0.99866 |
|  | Chiroptera | BM | 0.54739 | nested Shift | 0.35826 |
|  | Diprotodontia | BM | 0.57817 | nested EB Rate | 0.99999 |
|  | Primates | EB | 0.99999 | EB | 0.76861 |
|  | Rodentia | EB | 0.91835 | nested EB Rate | 0.99999 |
|  | Soricomorpha | EB | 0.6402 | nested EB Rate | 0.99999 |
| Mammalia Families | Bovidae | BM | 0.57451 | nested Shift | 0.73067 |
|  | Cricetidae | EB | 0.99999 | EB | 0.99755 |
|  | Muridae | BM | 0.53944 | nested EB Rate | 0.95925 |
|  | Phyllostomidae | EB | 0.99999 | nested EB Rate | 0.94645 |
|  | Pteropodidae | BM | 0.58245 | nested Shift | 0.69121 |
|  | Sciuridae | OU | 0.61768 | nested EB | 0.4698 |
|  | Soricidae | BM | 0.57815 | nested Shift | 0.72244 |
|  | Vespertilionidae | OU | 0.72765 | nested Shift | 0.36986 |
| Aves Orders | Accipitriformes | BM | 0.57855 | nested EB Rate | 0.96695 |
|  | Anseriformes | BM | 0.55474 | nested EB Rate | 0.9743 |
|  | Apodiformes | BM | 0.58727 | nested Shift | 0.62502 |
|  | Charadriiformes | BM | 0.58702 | nested Shift | 0.64844 |
|  | Galliformes | BM | 0.47165 | nested EB Rate | 0.82358 |
|  | Passeriformes | BM | 0.47165 | nested EB Rate | 0.82358 |
|  | Piciformes | BM | 0.51501 | nested OU | 0.30458 |
|  | Procellariiformes | BM | 0.51501 | nested OU | 0.30458 |
|  | Psittaciformes | BM | 0.49321 | nested Shift | 0.55379 |
|  | Strigiformes | BM | 0.5841 | nested Shift | 0.37559 |
| Aves Families | Accipitridae | BM | 0.54928 | nested Shift | 0.4711 |
|  | Anatidae | BM | 0.58633 | nested EB Rate | 0.43053 |
|  | Columbidae | BM | 0.57666 | nested EB Rate | 0.99999 |
|  | Cuculidae | EB | 0.56306 | nested EB Rate | 0.83337 |
|  | Emberizidae | BM | 0.53773 | nested EB Rate | 0.97546 |
|  | Furnariidae | EB | 0.8942 | EB | 0.35808 |
|  | Muscicapidae | BM | 0.58346 | nested EB | 0.44249 |
|  | Picidae | BM | 0.58311 | nested EB Rate | 0.51678 |
|  | Psittacidae | BM | 0.56744 | nested EB Rate | 0.49206 |
|  | Thamnophilidae | EB | 0.99998 | EB | 0.83369 |
|  | Thraupidae | BM | 0.58164 | nested EB Rate | 0.99855 |
|  | Trochilidae | BM | 0.58326 | nested EB Rate | 0.99701 |
|  | Turdidae | BM | 0.58978 | nested OU | 0.7548 |
|  | Tyrannidae | BM | 0.58192 | nested EB Rate | 0.99996 |
| Squamata | Agamidae | BM | 0.55598 | nested Shift | 0.71105 |
| Squamata | Colubridae | BM | 0.44204 | nested Shift | 0.74397 |
| Squamata | Lacertidae | BM | 0.58962 | nested Shift | 0.58294 |
| Squamata | Scincidae | OU | 0.49897 | nested Shift | 0.50681 |

**Supplementary Table S7.** The supported models with no AICc correction when body mass evolution is analysed at the whole-phylogeny level (BM, OU, and EB) compared to models when the initial mode of BM evolution can change within nested clades (nested EB, nested EB rate, nested OU, and nested Shift**).** All models were also applied individually to families, orders, and sub-orders with at least 100 species.

| ***Order*** | ***Families contained in nested shift*** |
| --- | --- |
| Artiodactyla | Bovidae partial |
| Carnivora | Eupleridae Herpestidae Nandiniidae Viverridae |
| Chiroptera | Emballonuridae Furipteridae Hipposideridae Megadermatidae Molossidae Mormoopidae Mystacinidae Myzopodidae Natalidae Noctilionidae Nycteridae Phyllostomidae Rhinolophidae Rhinopomatidae Thyropteridae Vespertilionidae |
| Diprotodontia | Acrobatidae Burramyidae Hypsiprymnodontidae Macropodidae Petauridae Phalangeridae Potoroidae Pseudocheiridae Tarsipedidae |
| Rodentia | Cricetidae Muridae Spalacidae |
| Soricomorpha | Soricidae partial |
|  |  |
| Accipitriformes | Accipitridae partial |
| Anseriformes | Anatidae partial |
| Apodiformes | Trochilidae |
| Passeriformes | Acanthizidae Acrocephalidae Aegithalidae Alaudidae Arcanatoridae Artamidae Bernieridae Bombycillidae Buphagidae Calcariidae Callaeidae Campephagidae Cardinalidae Certhiidae Cettiidae Chloropseidae Cinclidae Cisticolidae Climacteridae Cnemophilidae Coerebidae Conopophagidae Corcoracidae Corvidae Cotingidae Cracticidae Dasyornithidae Dicaeidae Dicruridae Donacobiidae Emberizidae Erythrocercidae Estrildidae Eurylaimidae Formicariidae Fringillidae Furnariidae Grallariidae Hirundinidae Hyliotidae Hypocoliidae Icteridae Incertae Sedis Irenidae Laniidae Leiothrichidae Locustellidae Macrosphenidae Malaconotidae Maluridae Melanocharitidae Melanopareiidae Meliphagidae Mimidae Mohouidae Monarchidae Motacillidae Muscicapidae Nectariniidae Neosittidae Nicatoridae Oriolidae Orthonychidae Pachycephalidae Panuridae Paradisaeidae Paramythiidae Pardalotidae Paridae Parulidae Passeridae Pellorneidae Petroicidae Peucedramidae Phylloscopidae Picathartidae Pipridae Pittidae Platysteiridae Ploceidae Pnoepygidae Polioptilidae Pomatostomidae Prionopidae Promeropidae Prunellidae Psophodidae Ptilonorhynchidae Pycnonotidae Regulidae Remizidae Rhinocryptidae Rhipiduridae Scotocercidae Sittidae Stenostiridae Sturnidae Sylviidae Tephrodornithidae Thamnophilidae Thraupidae Tichodromidae Timaliidae Tityridae Troglodytidae Turdidae Tyrannidae Vangidae Viduidae Vireonidae Zosteropidae |
| Piciformes | Capitonidae Indicatoridae Lybiidae Megalaimidae Picidae Ramphastidae Semnornithidae |
| Psittaciformes | Psittacidae partial |
| Strigiformes | Strigidae partial |

**Supplementary Table S8.** A summary of the families included within the nested shift models. Families marked as *partial* indicate only a subset of the family was included in the nested shift model.

| Mammalia Orders | | | | | | | |
| --- | --- | --- | --- | --- | --- | --- | --- |
|  | m.sig | c.var | s.var | s.asr | s.hgt | d.cdf | model |
| Artiodactyla | 0.917 | 0.000 | 0.336 | 0.773 | 0.334 | 0.026 | nested EB rate |
| Carnivora | **0.981** | **0.498** | **0.320** | **0.977** | **0.617** | **0.158** | **nested shift** |
| Chiroptera | 0.991 | 0.000 | 0.000 | 0.020 | 0.258 | 0.294 | nested EB rate |
| Diprotodontia | 0.927 | 0.000 | 0.468 | 0.046 | 0.296 | 0.100 | nested EB rate |
| Primates | 0.893 | 0.000 | 0.637 | 0.006 | 0.324 | 0.020 | EB |
| Rodentia | 0.961 | 0.000 | 0.354 | 0.000 | 0.000 | 0.000 | nested EB rate |
| Soricomorpha | 0.939 | 0.000 | 0.623 | 0.857 | 0.701 | 0.014 | nested EB |
| Aves Orders | | | | | | | |
| Accipitriformes | **0.907** | **0.418** | **0.773** | **0.066** | **0.490** | **0.851** | **nested EB rate** |
| Anseriformes | **0.971** | **0.296** | **0.507** | **0.779** | **0.939** | **0.376** | **nested EB rate** |
| Apodiformes | 0.931 | 0.000 | 0.849 | 0.573 | 0.769 | 0.328 | nested OU |
| Charadriiformes | 0.953 | 0.000 | 0.501 | 0.412 | 0.897 | 0.126 | OU |
| Galliformes | 0.877 | 0.002 | 0.795 | 0.084 | 0.162 | 0.148 | nested shift |
| Passeriformes | 0.969 | 0.000 | 0.757 | 0.000 | 0.296 | 0.000 | nested EB rate |
| Piciformes | **0.921** | **0.422** | **0.280** | **0.346** | **0.921** | **0.535** | **OU** |
| Procellariiformes | 0.931 | 0.002 | 0.985 | 0.286 | 0.691 | 0.026 | EB |
| Psittaciformes | 0.987 | 0.000 | 0.595 | 0.004 | 0.801 | 0.472 | nested EB rate |
| Strigiformes | 0.915 | 0.000 | 0.905 | 0.066 | 0.865 | 0.220 | nested EB rate |
| Mammalia Families | | | | | | | |
| Bovidae | 0.939 | 0.000 | 0.170 | 0.336 | 0.226 | 0.026 | nested EB rate |
| Cricetidae | 0.947 | 0.000 | 0.476 | 0.162 | 0.527 | 0.016 | nested EB rate |
| Muridae | 0.947 | 0.424 | 0.128 | 0.002 | 0.835 | 0.390 | nested EB rate |
| Phyllostomidae | **0.973** | **0.853** | **0.551** | **0.843** | **0.843** | **0.545** | **nested EB rate** |
| Pteropodidae | 0.917 | 0.000 | 0.166 | 0.010 | 0.775 | 0.132 | nested EB rate |
| Sciuridae | 0.923 | 0.000 | 0.949 | 0.412 | 0.783 | 0.048 | nested shift |
| Soricidae | 0.901 | 0.080 | 0.012 | 0.338 | 0.867 | 0.715 | nested EB |
| Vespertilionidae | 0.939 | 0.000 | 0.170 | 0.336 | 0.226 | 0.026 | nested EB rate |
| Aves Families | | | | | | | |
| Accipitridae | **0.933** | **0.240** | **0.949** | **0.070** | **0.619** | **0.981** | **nested EB rate** |
| Anatidae | **0.919** | **0.298** | **0.104** | **0.753** | **0.723** | **0.118** | **nested EB rate** |
| Columbidae | **0.941** | **0.444** | **0.811** | **0.340** | **0.208** | **0.402** | **nested EB rate** |
| Cuculidae | **0.877** | **0.290** | **0.120** | **0.110** | **0.242** | **0.507** | **nested OU** |
| Emberizidae | 0.927 | 0.196 | 0.440 | 0.000 | 0.697 | 0.501 | nested shift |
| Furnariidae | 0.983 | 0.002 | 0.895 | 0.128 | 0.611 | 0.130 | nested shift |
| Muscicapidae | 0.907 | 0.142 | 0.354 | 0.010 | 0.416 | 0.603 | nested EB |
| Picidae | **0.937** | **0.707** | **0.276** | **0.601** | **0.583** | **0.633** | **OU** |
| Psittacidae | 0.935 | 0.000 | 0.643 | 0.010 | 0.797 | 0.084 | nested EB rate |
| Thamnophilidae | 0.909 | 0.002 | 0.342 | 0.014 | 0.216 | 0.152 | EB |
| Thraupidae | 0.913 | 0.000 | 0.202 | 0.086 | 0.989 | 0.204 | nested EB rate |
| Trochilidae | 0.965 | 0.002 | 0.649 | 0.619 | 0.334 | 0.256 | OU |
| Turdidae | 0.871 | 0.024 | 0.406 | 0.222 | 0.010 | 0.667 | nested OU |
| Tyrannidae | 0.947 | 0.000 | 0.184 | 0.036 | 0.873 | 0.274 | nested EB rate |
| Squamata Families | | | | | | | |
| Agamidae | **0.959** | **0.112** | **0.973** | **0.723** | **0.795** | **0.330** | **nested shift** |
| Colubridae | 0.907 | 0.000 | 0.256 | 0.084 | 0.004 | 0.064 | nested shift |
| Lacertidae | 0.959 | 0.002 | 0.891 | 0.020 | 0.100 | 0.747 | nested EB |
| Scincidae | 0.903 | 0.020 | 0.250 | 0.010 | 0.476 | 0.979 | nested EB |

**Supplementary Table S9. Model adequacy of the best relative model tested using six metrics described by Pennell *et al.* (2015).** Models have better adequacy when applied to smaller phylogenies (i.e, to families), but models fit poorly to Squamata particularly in their ability to detect rate heterogeneity. Models that are adequate according to all six metrics are shown in **bold**.

|  |  | ***Multi-rate BM model*** | | | | | ***Multi-rate OU model*** | | | | |
| --- | --- | --- | --- | --- | --- | --- | --- | --- | --- | --- | --- |
| ***Clade*** | **Nested *Model*** | ***slope* (*b_1_*)** | ***p value*** | ***slope* (*b_1_*) *(outliers removed)*** | ***p value (outliers removed)*** | ***Robust linear regression slope* (*b_1_*)** | ***slope* (*b_1_*)** | ***p value*** | ***slope* (*b_1_*) *(outliers removed)*** | ***p value (outliers removed)*** | ***Robust linear regression slope* (*b_1_*)** |
| **Artiodactyla** | *nested EB Rate* | -0.0395 | 0.0242 | -0.0395 | 0.0242 | -0.0344 | -3.1084 | 0.0149 | -3.1084 | 0.0149 | -2.6791 |
| **Bovidae** | *nested EB Rate* | -0.0397 | 0.024 | -0.0397 | 0.024 | -0.0344 | -1.2755 | 0.0173 | -1.2755 | 0.0173 | -1.1405 |
| **Carnivora** | *nested EB Rate* | -0.0086 | 0.6512 | 0.0021 | 0.9041 | 0.0023 | 10.3627 | 0.0116 | -1.5706 | 0.2599 | 0.1724 |
| **Chiroptera** | *nested EB Rate* | 0.0037 | 0.5158 | 0.013 | 0.0012 | 0.0119 | -0.4179 | 0.3316 | 0.2850 | 0.3311 | 0.2770 |
| **Diprotodontia** | *nested EB Rate* | -0.0315 | 0.0311 | -0.0315 | 0.0311 | -0.0307 | 1.7705 | 0.3873 | -1.7612 | 0.0348 | -1.3351 |
| **Muridae** | *nested EB Rate* | -0.0739 | 0.0007 | -0.0548 | 0.0007 | -0.0279 | -2.4935 | 0.0258 | -2.6890 | 0.0013 | -1.1432 |
| **Pteropodidae** | *nested EB Rate* | 0.0042 | 0.8061 | -0.0075 | 0.6497 | -0.001 | -0.0608 | 0.8931 | -0.3879 | 0.3677 | -0.3430 |
| **Rodentia** | *nested EB Rate* | -0.0923 | 0.0001 | -0.0296 | 0.0008 | -0.0448 | -6.8095 | 0.0001 | -2.1118 | 0.0050 | -3.4240 |
| **Soricidae** | *nested EB* | -0.0742 | 0.018 | -0.0508 | 0.0024 | -0.0554 | -2.8075 | 0.0753 | -1.6337 | 0.0649 | -2.0667 |
| **Accipitridae** | *nested EB Rate* | 0.0015 | 0.9449 | 0.0137 | 0.3043 | 0.0149 | 0.1814 | 0.8904 | 0.9202 | 0.2508 | 0.8647 |
| **Accipitriformes** | *nested EB Rate* | 0.001 | 0.9635 | 0.0132 | 0.323 | 0.0145 | 0.1715 | 0.9260 | 1.2117 | 0.2774 | 1.1608 |
| **Anseriformes** | *nested EB Rate* | 0.0298 | 0.1119 | -0.0043 | 0.7711 | 0.0022 | 0.5723 | 0.6210 | 0.1263 | 0.8925 | 0.2197 |
| **Apodiformes** | *nested EB Rate* | 0.007 | 0.6564 | 0.0085 | 0.4831 | 0.0016 | -1.3249 | 0.2200 | -0.6596 | 0.4254 | -1.0785 |
| **Charadriiformes** | *nested EB Rate* | -0.0244 | 0.3422 | -0.0312 | 0.1862 | -0.0173 | -0.3884 | 0.8324 | -0.8880 | 0.5949 | 0.0676 |
| **Columbidae** | *nested EB Rate* | -0.0201 | 0.154 | -0.0098 | 0.3242 | -0.0116 | -0.1217 | 0.8365 | 0.2823 | 0.5248 | -0.1061 |
| **Muscicapidae** | *nested EB Rate* | -0.1129 | 0.0227 | -0.0301 | 0.3342 | -0.0275 | -1.1276 | 0.1624 | 0.1787 | 0.7415 | 0.3156 |
| **Passeriformes** | *nested EB Rate* | -0.0193 | 0 | -0.0055 | 0.0272 | -0.0086 | -1.6531 | 0.0000 | -0.6473 | 0.0004 | -0.8531 |
| **Picidae** | *nested EB Rate* | -0.0329 | 0.2507 | -0.0274 | 0.2804 | -0.0248 | 0.3257 | 0.6872 | -0.1385 | 0.8455 | -0.2702 |
| **Piciformes** | *nested EB Rate* | -0.0443 | 0.0376 | -0.0223 | 0.1412 | -0.0249 | -1.0621 | 0.3708 | 0.1856 | 0.8237 | 0.1557 |
| **Psittacidae** | *nested EB* | -0.0752 | 0.0715 | -0.0752 | 0.0715 | -0.0673 | -2.3792 | 0.0229 | -2.3792 | 0.0229 | -2.1913 |
| **Psittaciformes** | *nested EB* | -0.069 | 0.0679 | -0.0318 | 0.2426 | -0.0474 | -6.3595 | 0.0020 | -4.2364 | 0.0022 | -4.1892 |
| **Strigiformes** | *nested EB Rate* | -0.1015 | 0.003 | -0.0221 | 0.1943 | -0.0412 | -2.8184 | 0.4323 | -6.4008 | 0.0177 | -1.3630 |
| **Thraupidae** | *nested EB Rate* | 0.0376 | 0.3613 | 0.0156 | 0.6024 | 0.0079 | 0.7579 | 0.4300 | 0.2686 | 0.7119 | 0.0888 |
| **Trochilidae** | *nested EB Rate* | 0.0169 | 0.7017 | 0.0555 | 0.0621 | 0.0324 | -1.1605 | 0.3623 | -0.0495 | 0.9537 | -0.4829 |
| **Tyrannidae** | *nested EB Rate* | -0.0094 | 0.7649 | 0.0135 | 0.5791 | 0.0075 | -1.0107 | 0.2857 | -0.3424 | 0.6444 | -0.2608 |
| **Lacertidae** | *nested EB* | -0.0354 | 0.026 | -0.0037 | 0.5811 | -0.0074 | -2.3327 | 0.0939 | 0.4286 | 0.4909 | -0.0644 |
| **Scincidae** | *nested EB* | -0.0545 | 0.1674 | -0.0337 | 0.0765 | -0.0049 | -4.6262 | 0.0358 | -0.4711 | 0.5360 | -0.9516 |

**Supplementary Table S10. Node height tests on the estimated log-scaled standardised independent phylogenetic contrasts expected under multi-rate Brownian motion and OU models respectively.** The analyses were performed on subclades that supported *nested EB* models**.** A negative slope is indicative of an exponential decrease in the difference between standardised contrasts through time which is the pattern expected under an early burst model.

|  | Branch rates in EB clade lower than rest of the tree | Rate of branch leading to the crown clade higher than branch rates in EB clade |
| --- | --- | --- |
| **Artiodactyla** | ***p < 0.001*** | ***p < 0.001*** |
| **Bovidae** | ***p < 0.001*** | ***p < 0.001*** |
| **Carnivora** | ***p < 0.001*** | ***p < 0.001*** |
| **Chiroptera** | ***p < 0.001*** | ***p < 0.001*** |
| Diprotodontia | 0.168490521 | 0.705028257 |
| Muridae | 1 | 1 |
| Pteropodidae | 1 | 1 |
| **Rodentia** | ***p < 0.001*** | ***p < 0.001*** |
| **Soricidae** | ***p < 0.001*** | ***p < 0.001*** |
|  |  |  |
| Accipitridae | 1 | 1 |
| Accipitriformes | 1 | 1 |
| Anseriformes | 1 | 1 |
| **Apodiformes** | ***p < 0.001*** | ***p < 0.001*** |
| Charadriiformes | 1 | 1 |
| Columbidae | 0.237056611 | *p < 0.001* |
| Muscicapidae | *p < 0.001* | 1 |
| **Passeriformes** | ***p < 0.001*** | ***p < 0.001*** |
| Picidae | 0.255430538 | 1 |
| Piciformes | 0.999994816 | 1 |
| **Psittacidae** | ***p < 0.001*** | ***p < 0.001*** |
| **Psittaciformes** | ***p < 0.001*** | ***p < 0.001*** |
| Strigiformes | *p < 0.001* | 0.995249458 |
| Thraupidae | 0.999770962 | 0.999832129 |
| Trochilidae | 1 | 0.999864444 |
| Tyrannidae | 1 | 1 |
|  |  |  |
| Lacertidae | *p < 0.001* | 0.999999998 |
| **Scincidae** | ***p < 0.001*** | ***p < 0.001*** |

**Supplementary Table S11. A summary of rates from the *auteur* analyses for trees that show the highest support for the *nested EB and nested EB rate* models.** The first column shows the *p* value of a Wilcoxon test indicating whether the branch rates in the clade categorised as undergoing an EB process are lower than the rest of the tree, and if the rate on the branch leading to the most common ancestor of the EB clade has a higher rate than the rates within the EB clade.

| Artiodactyla | 180 |
| --- | --- |
| Bovidae | 115 |
| Carnivora | 232 |
| Chiroptera | 742 |
| Cricetidae | 447 |
| Diprotodontia | 101 |
| Muridae | 402 |
| Phyllostomidae | 122 |
| Primates | 208 |
| Pteropodidae | 130 |
| Rodentia | 1429 |
| Sciuridae | 236 |
| Soricidae | 186 |
| Soricomorpha | 217 |
| Vespertilionidae | 228 |
| Accipitridae | 142 |
| Accipitriformes | 151 |
| Anatidae | 136 |
| Anseriformes | 139 |
| Apodiformes | 235 |
| Charadriiformes | 216 |
| Columbidae | 124 |
| Cuculidae | 104 |
| Emberizidae | 104 |
| Furnariidae | 188 |
| Galliformes | 157 |
| Muscicapidae | 148 |
| Passeriformes | 3179 |
| Picidae | 125 |
| Piciformes | 216 |
| Procellariiformes | 101 |
| Psittacidae | 196 |
| Psittaciformes | 214 |
| Strigiformes | 101 |
| Thamnophilidae | 150 |
| Thraupidae | 270 |
| Trochilidae | 210 |
| Turdidae | 110 |
| Tyrannidae | 258 |
| Agamidae | 111 |
| Colubridae | 119 |
| Lacertidae | 113 |
| Scincidae | 187 |

**Supplementary Table S12.** The number of species included in each phylogeny in the analysis
